# Whole-genome sequencing reveals *KRTAP1-1* as a novel genetic variant associated with antidepressant treatment outcomes

**DOI:** 10.1038/s41598-021-83887-6

**Published:** 2021-02-25

**Authors:** Jong-Ho Park, Shinn-Won Lim, Woojae Myung, Inho Park, Hyeok-Jae Jang, Seonwoo Kim, Min-Soo Lee, Hun Soo Chang, DongHo Yum, Yeon-Lim Suh, Jong-Won Kim, Doh Kwan Kim

**Affiliations:** 1 Department of Health Sciences and Technology, SAIHST, Sungkyunkwan University, Seoul, Korea; 2 Clinical Genomics Center, Samsung Medical Center, Seoul, Korea; 3 Department of Neuropsychiatry, Seoul National University Bundang Hospital, Seongnam, Korea; 4 Precision Medicine Center, Gangnam Severance Hospital, Yonsei University College of Medicine, Seoul, Korea; 5 Statistics and Data Center, Research Institute for Future Medicine, Samsung Medical Center, Seoul, Korea; 6 Department of Psychiatry, College of Medicine, Korea University, Seoul, Korea; 7 Soonchunhyang Medical Institute, College of Medicine, Soonchunhyang University, Asan, Korea; 8 Department of Pathology, Samsung Medical Center, Sungkyunkwan University School of Medicine, Seoul, Korea; 9 Department of Laboratory Medicine and Genetics, Samsung Medical Center, Sungkyunkwan University School of Medicine, 81 Irwon-ro, Gangnam-gu, Seoul, 135-710 Korea; 10 Department of Psychiatry, Samsung Medical Center, Sungkyunkwan University School of Medicine, 81 Irwon-ro, Gangnam-gu, Seoul, 135-710 Korea

**Keywords:** Genetics, Biomarkers, Diseases, Molecular medicine

## Abstract

Achieving remission following initial antidepressant therapy in patients with major depressive disorder (MDD) is an important clinical result. Making predictions based on genetic markers holds promise for improving the remission rate. However, genetic variants found in previous genetic studies do not provide robust evidence to aid pharmacogenetic decision-making in clinical settings. Thus, the objective of this study was to perform whole-genome sequencing (WGS) using genomic DNA to identify genetic variants associated with the treatment outcomes of selective serotonin reuptake inhibitors (SSRIs). We performed WGS on 100 patients with MDD who were treated with escitalopram (discovery set: 36 remitted and 64 non-remitted). The findings were applied to an additional 553 patients with MDD who were treated with SSRIs (replication set: 185 remitted and 368 non-remitted). A novel loss-of-function variant (rs3213755) in keratin-associated protein 1–1 (*KRTAP1-1*) was identified in this study*.* This rs3213755 variant was significantly associated with remission following antidepressant treatment (*p* = 0.0184, OR 3.09, 95% confidence interval [CI] 1.22–7.80 in the discovery set; *p* = 0.00269, OR 1.75, 95% CI 1.22–2.53 in the replication set). Moreover, the expression level of *KRTAP1-1* in surgically resected human temporal lobe samples was significantly associated with the rs3213755 genotype. WGS studies on a larger sample size in various ethnic groups are needed to investigate genetic markers useful in the pharmacogenetic prediction of remission following antidepressant treatment.

## Introduction

Major depressive disorder (MDD) is one of the most common debilitating diseases that has major public health and economic consequences^1^. Antidepressant medication provides effective treatment for patients with MDD^2,3^. However, initial antidepressant treatment can fail in 30–40% of patients^4^. Remission following depression treatment has clinical importance because it is associated with functional recovery and a better prognosis^5^. Moreover, antidepressant therapy takes time to achieve an effect, usually 2–4 weeks. Currently, it is not possible to predict which drug will be the most effective for an individual patient. There is a great deal of motivation on the professional side to provide better treatments: genetic prediction represents a good starting point for such improvements^5^.

The effects of antidepressants were demonstrated to have genetic variability^6–8^. A single nucleotide polymorphism (SNP)-based heritability could explain up to 42% of the variance in antidepressant responses^9^. However, genetic variants found in previous genome-wide association studies (GWASs) on the antidepressant response do not provide robust evidence to aid pharmacogenetic decision-making in clinical settings^10–16^. Most whole-sample analyses of genetic variants did not attain statistical significance at the genome-wide level. Additionally, there is no genetic variants robustly replicated, and none of these previous antidepressant pharmacogenetics studies identified significant SNPs related to remission^10–14^. GWASs typically focused on common variants that represent only a small fraction of all variants related to the antidepressant mechanism of action. Consequently, the “common disease–common variant” hypothesis based on GWASs has not fulfilled expectations in the field of pharmacogenetics associated with depression^17^.With the recent advances in genetic technology, whole-genome sequencing (WGS) has offers multiple advantages in genomic variant discovery^18^. WGS can provide information regarding all genetic variants—rare variants, loss of function (LOF), functional intronic variants, and structural variants. Significant outcomes from studies on these variants can provide useful information to pinpoint causal genes and their associated pathways. LOF variants have been reported to significantly affect protein function and, as a result, depression^19,20^ and pharmacogenomic traits of antidepressants^21^. There have been several pharmacogenetic studies on antidepressant treatment response using genetic sequencing, including an exome sequencing^8,22,23^, and a targeted sequencing study^24^. Despite the advantages of WGS, studies with sufficient sample sizes have not yet been reported due to high cost and high computational resources^25^.

Therefore, here, we conducted a preliminary WGS study to identify genetic variants associated with remission following antidepressant treatment in a discovery set with a small sample size (n = 100) along with a replication set (n = 553). Although it is difficult to obtain robust results using a small sample size, in this study, we tried to determine the applicability of WGS in this field. Our hypothesis was that genetic germline variants found using WGS might be associated with remission following antidepressant treatment with selective serotonin reuptake inhibitors (SSRIs).

## Results

### Subject characteristics

The subjects’ clinical and demographic characteristics of the subjects are shown in Table 1. The subjects were mostly elderly individuals (median age 68 and 62 years for the discovery and replication sets, respectively) who were experiencing their second or later MDD episodes. Approximately one-fifth of these patients had a family history of depression. The pretreatment Hamilton scale for depression (HAM-D) scores indicated moderate to severe depression (median score 19 and 20 for the discovery and replication sets, respectively). The observed remission rates were above 30% for both the discovery and replication sets. Age and age at onset differed between the discovery and replication sets. Family history of depression, number of episodes, and baseline HAM-D score in the discovery set and sex and baseline HAM-D score in the replication set were associated with remission following antidepressant treatment.

**Table 1:** Clinical and demographic characteristics of the completer cohorts.

Characteristics	Discovery set (*n* = 100)				Replication set (*n* = 553)				*p* (Discovery set *vs.* replication set)
	Escitalopram-treated group				SSRI-treated group				
	Total	With remission	Without remission	*p*	Total	With remission	Without remission	*p*	
Remission rate	36 (36.0%)				185 (33.5%)				0.62
Response rate	60 (60.0%)				299 (54.1%)				0.27
Sex, female (%)^a^	74 (74.0%)	25 (69.4%)	49 (76.6%)	0.44	418 (75.6%)	152 (82.2%)	266 (72.3%)	0.01	0.74
Age, year^b^	68 (59–72)	67 (61.5–73.5)	69 (58–72)	0.80	62 (51–69)	61 (48–68)	62 (52–70)	0.15	< 0.0001
Family history of depression (%)^a^	16 (16.0%)	2 (5.6%)	14 (21.9%)	0.03	108 (19.5%)	33 (17.8%)	75 (20.4%)	0.48	0.41
Number of episodes^b^	2 (1–2.5)	1 (1–2)	2 (1–3)	0.01	2 (1–2)	2 (1–2)	2 (1–3)	0.11	0.51
Age at onset, year^b^	61 (48–69)	63 (52–70)	60 (42.5–68.5)	0.09	54 (40–64)	53 (40–63)	54.5 (41–64)	0.34	0.002
HAM-D baseline^b^	19 (17–22)	18 (16–20)	21 (18–24)	< 0.0001	20 (17–23)	19 (17–22)	21 (18–23)	< 0.0001	0.16

*HAM-D* Hamilton depression rating score, *SSRIs* selective serotonin reuptake inhibitors.

^a^Chi-square test was performed.

^b^Wilcoxon’s rank sum test was performed. Ranges shown are inter-quartile ranges.

### WGS characteristics

We conducted WGS for 100 patients with MDD treated with SSRIs. The average depth showed a 31.8 × coverage of the whole genome. All patients with MDD in the discovery set were confirmed to be ethnically homogeneous; thus, there was no population stratification (Supplementary Fig. S1). The average transition/transversion (Ts/Tv) ratio, computed as a quality control (QC) parameter in the whole genome using the SNV data, was 1.97. This value is generally around 2.0 based on previous WGS reports^26^. By applying a QC filter (as mentioned in the “Methods”), the final call set contained 13,318,214 variants comprising 9,736,393 single nucleotide variations (SNVs) and 3,581,821 insertions and deletions (INDELs). Of these 13,318,214 variants, 8,871,350 SNVs and 1,896,224 INDELs matched with variants from the dbSNP 138 database. The number of novel variants that did not match with dbSNP was 865,043 for SNVs (8.88%) and 1,685,597 for INDELs (47.06%). Because of the exclusion of variants detected in less than two samples from the association analysis, we observed a lower novel variants rate (calculated as the number of variants that matched with dbSNP/the number of variants in the final set) in our final variant set. We first carried out genome-wide association analysis using the final variant set, 13,318,214 variants, called from WGS in the discovery set. The association test results revealed no variant with a genome-wide significance threshold of 5 × 10^–8^ when considering both response to and remission following antidepressant treatment. However, 18 variants (17 SNVs and 1 INDEL) and another set of 18 variants (16 SNVs and 2 INDELs) showed a *P* value < 10^–5^ for remission following antidepressant treatment (Supplementary Table S1) and response to antidepressant treatment, respectively (Supplementary Table S2).

### Identification of novel candidate variants associated with remission following antidepressant treatment in the discovery set

**Figure 1:**
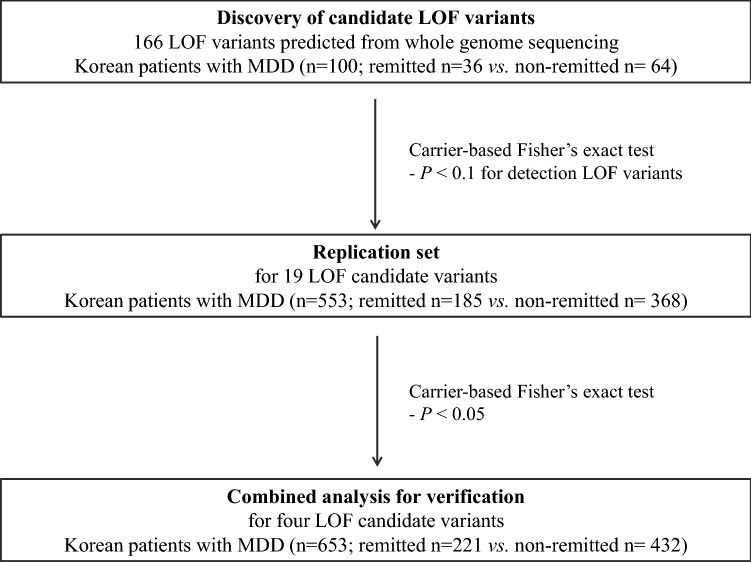
Schematic workflow.

For WGS analysis, we focused on the penetrating LOF mutations known to disrupt protein function (Fig. 1) rather than common signals. We identified four SNVs predicted to be stop-gained SNVs among the variants (Table 2). The four SNVs (rs1476860, rs3213755, rs139506139, and rs877346) resided close to the genes *OR1B1*, keratin-associated protein 1–1 (*KRTAP1-1*), *SRRM5,* and *KRTAP13-2*, respectively. As a representative SNV, rs3213755 was found to be located in *KRTAP1-1* as an LOF variant that could block gene translation and induce the disruption of protein function; it showed the most significant association with remission following antidepressant treatment in the discovery set (*p* = 0.0184, OR 3.09, 95% confidence interval [CI] 1.22–7.80). Additionally, rs1476860 (*p* = 0.077, OR 4.33, 95% CI 0.92–20.43), rs139506139 (*p* = 0.044, OR 0.07, 95% CI 0.0037–1.48), and rs877346 (*p* = 0.055, OR 2.49, 95% CI 1.01–6.12) were selected as candidate variants associated with remission in the discovery set.

**Table 2:** Candidate loss-of-function variants associated with remission following SSRI treatment in patients with MDD (discovery set, *n* = 100; replication set, *n* = 553).

Chromosome	SNP	Position^†^	Gene	Minor/major allele	Cohort^a^	MAF in non-remitted	MAF in remitted	*p* value^‡^	OR (CI, 95%)^‡^
9	rs1476860	125391241	*OR1B1*	A/G	Discovery	0.391	0.333	0.077*	4.33 (0.92–20.43)*
					Replication	0.370	0.370	0.517*	1.21 (0.72–2.04)*
					Combined	0.373	0.364	0.155*	1.44 (0.88–2.35)*
17	rs3213755	39197499	*KRTAP1-1*	A/G	Discovery	0.234	0.125	0.0184	3.09 (1.22–7.80)
					Replication	0.331	0.228	0.00269	1.75 (1.22–2.53)
					Combined	0.316	0.211	0.00017	1.90 (1.36–2.67)
19	rs139506139	44117804	*SRRM5*	T/C	Discovery	0	0.042	0.044	0.07 (0.0037–1.48)
					Replication	0.012	0.022	0.296	0.55 (0.21–1.46)
					Combined	0.010	0.025	0.054	0.41 (0.17–1.00)
21	rs877346	31744127	*KRTAP13-2*	T/A	Discovery	0.266	0.125	0.055	2.49 (1.01–6.12)
					Replication	0.190	0.160	0.292	1.23 (0.84–1.80)
					Combined	0.201	0.155	0.080	1.37 (0.97–1.95)

*MAF* minor allele frequency, *OR* odds ratio, *CI* confidence interval, *SNP* single nucleotide polymorphism, *NA* not available.

^†^Physical position based on human reference genome build hg 19 (GRCh37).

^‡^*p* value and OR were calculated using a dominant disease model. The dominant allele is a test for the minor allele.

*p value was calculated using a recessive disease model.

^a^In the discovery set, remitted, *n* = 36; non-remitted, *n* = 64; in the replication set, remitted, *n* = 185; non-remitted, *n* = 368.

### Validation of candidate variants associated with remission following antidepressant treatment in the replication set

A focused replication study was performed on these four candidate SNVs (rs1476860, rs3213755, rs139506139, and rs877346; Table 2). Of the four SNVs, rs3213755 showed a significant association with remission (*p* = 0.0027, OR 1.75, 95% CI 1.22–2.53]) in the replication set (*n* = 553). In a combined analysis of both the discovery and replication sets, rs3213755 showed a significant association with remission (*p* = 0.00017, OR 1.90, 95% CI 1.36–2.67). The frequency of the rs3213755 A allele in the non-remission group from the discovery and replication sets was higher than that in the remission group (0.32 *vs.* 0.21 in the combined set). Notably, when only one SSRI (escitalopram) was considered, rs3213755 also showed a significant association (*p* = 0.00014, OR 2.75, 95% CI 1.63–4.65) in the combined set (Supplementary Table S3). In the multivariate analysis, considering confounding factors, subjects possessing the LOF allele (A) of rs3213755 had a lower probability of achieving remission following antidepressant treatment, which was expected. The association between rs3213755 and remission after SSRI treatment was robust after adjusting for variables including age, sex, family history of depression, number of episodes, age of onset, and baseline HAM-D score in the replication set (*p* = 0.0055, OR 1.71, 95% CI 1.17–2.49). However, rs1476860 (*p* = 0.517), rs139506139 (*p* = 0.296), and rs877346 (*p* = 0.292) did not show a significant association with remission after SSRI treatment in the replication set.

### Co-expressed genes associated with *KRTAP1-1* from the in silico analysis and quantification in human brain tissue

**Figure 2:**
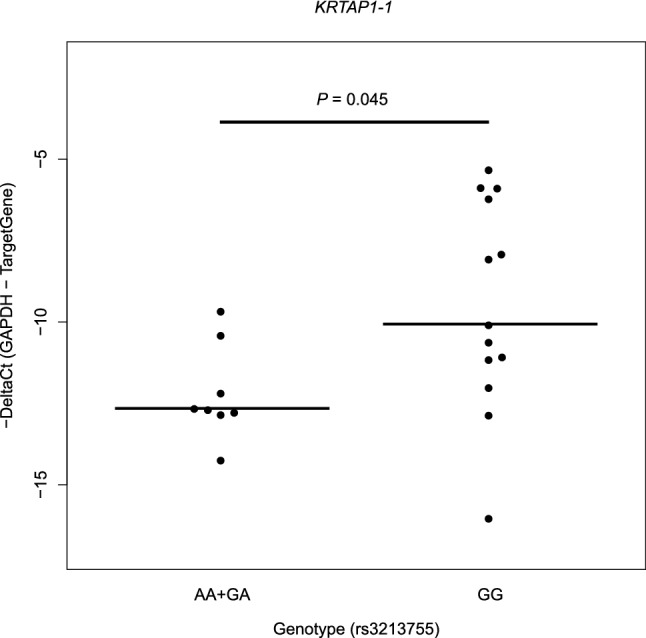
The loss-of-function variant, rs3213755, leads to low expression of the *KRTAP1-1* gene in brain tissues. Expression levels are presented as minus ΔCt (*GAPDH* Ct value—TargetGene Ct value). Statistical analysis was performed using Wilcoxon’s rank-sum test. The association test was considered as a dominant model for the minor allele (A). Statistical analysis was performed using Wilcoxon’s rank-sum test. One sample with extremely high expression was excluded from the statistical analysis as an outlier.

To gain further information on the function and associated pathways of *KRTAP1-1*, we additionally identified 20 genes related to *KRTAP1-1* from the GeneMANIA database, reflecting gene–gene interactions, co-expression patterns, and protein domains (Supplementary Fig. S2). Among these 20 genes, three genes (*NTF3*, *GRIA4,* and *ARG2*) were chosen based on functional evidence related to drug targets, neurological functions, and expression profiles in brain tissues. Finally, we quantified the expression of the four genes (*KRTAP1-1*, *NTF3*, *GRIA4,* and *ARG2*) in 22 temporal lobe tissue samples using qPCR. The results showed that all three genes (*NTF3*, *GRIA4,* and *ARG2*) along with *KRTAP1-1* were expressed in the temporal lobe tissues of the human brain. The LOF variant, rs3213755, with the A allele was directly and significantly associated with the lower expression of the *KRTAP1-1* gene in brain tissues (*p* = 0.045, Fig. 2). No significant association between the genotype and clinical characteristics of the subjects such as age or sex, was observed in this expression analysis (*p* > 0.05). Additionally, *NTF3* expression was highly positively correlated with *KRTAP1-1* expression in brain tissues based on Pearson’s correlation analysis (r = 0.84, *p* = 1.04 × 10^–6^, Supplementary Fig. S3).

### Quasi-replication and replication of previous genetic studies on the antidepressant response

Among the top 100 SNPs reported in previous GWASs on the antidepressant response^15^ in Korean populations (Supplementary Table S4), 11 variants showed significant associations with remission following SSRI treatment in our WGS results in the quasi-replication analysis (nominal threshold *p* < 0.05). Of these top 100 SNPs, 75 (75.0%) showed an identical direction to the previous GWAS and our WGS results. In the replication analysis, 27 SNPs showed significant associations (nominal threshold *p* < 0.05) with a response to SSRI treatment in our WGS results. Additionally, we obtained 90 (90.0%) variants with a concordance of direction in both studies. No variant was replicated in a previous candidate genetic study that investigated neurotransmitter-related genes (Supplementary Table S5)^27^. Among the SNPs reported in a previous meta-analyses of the antidepressant response in populations of European ancestry (*p* < 0.0001)^28^, only two SNPs (rs7597171 and rs2516808) were associated with remission, whereas three SNPs (rs7597171, rs943347, and rs2826852) were associated with a response in our WGS study (Supplementary Table S6).

### Association between candidate variants and the antidepressant response in the discovery and replication sets

The association between rs3213755 and the antidepressant response to SSRI treatment was not significant in the discovery set (*p* = 0.30, OR 1.64, 95% CI 0.72–3.72); it showed a significant association in the replication set (*p* = 0.00041, OR 1.86, 95% CI 1.32–2.61). Additionally, three SNVs, rs115678527 (*p* = 0.081, OR 0.12, 95% CI 0.0067–2.32), rs7021123 (*p* = 0.078, OR 0.10, 95% CI 0.0057–1.89), and rs1476860 (*p* = 0.096, OR 2.61, 95% CI 0.85–8.04), were identified as candidates associated with the antidepressant response in the discovery set. However, these SNVs (rs115678527; *p* = 1.00, rs7021123; *p* = 0.81, and rs1476860; *p* = 0.71) did not show a significant association with the response after SSRI treatment in the replication set (Supplementary Table S7).

## Discussion

We identified a novel LOF variant in *KRTAP1-1*, keratin-associated protein 1–1, that is associated with outcome of SSRI antidepressant therapy in patients with MDD. This variant, rs3213755, was identified using WGS. Its association with the treatment outcome of SSRI antidepressant therapy was validated in an independent set of patients with MDD (Table 1, Supplementary Table S8). The functional relevance between this variant and its expression was also evaluated in human brain tissues. Our results suggest that rs3213755 is an important variant associated with *KRTAP1-1* knock-out and that it is related to remission following antidepressant treatment.

A null mutation in *KRTAP1-1* could be caused by rs3213755; it can also be caused by additional LOF variants located in other coding regions. However, the only type of inactivating variant, rs3213755, known to produce a gene product with a minor or no function, was identified using WGS in the discovery set. We confirmed that the existence of the A allele of rs3213755 directly reduced the expression of *KRTAP1-1* in an in vitro experiment using human brain tissues (Fig. 2). Thus, *KRTAP1-1* may contribute as a mediator of the treatment outcome of remission following antidepressant therapy.

*KRTAP1-1*, a member of the keratin-associated protein (KAP) family located on chromosome 17q21.2, and encodes for a protein that forms a matrix of keratin intermediate filaments. *KRTAP1-1*′s function in the antidepressant effect has not been extensively investigated. Furthermore, its biological relevance in the human brain has not been systemically elucidated. However, although relatively low-expressed transcripts have not been detected in database such as Genotype-Tissue Expression (GTEx) based on RNA-sequencing, they can be measured using a more sensitive RT-qPCR method^29,30^. Accordingly, we investigated whether the genes co-expressed with this gene were associated with depression or the action mechanism of antidepressants. The *KRTAP1-1* transcript expression was highly correlated with *NTF3* expression in the temporal lobe of the human brain (Supplementary Fig. S2). Notably, the co-expressed gene, *NTF3*, can regulate cellular proliferation and apoptosis in keratinocytes^31^. *NTF3* is widely distributed in the cerebral hippocampus. It regulates neuronal development and promotes hippocampal plasticity^32^. A previous study using a mouse depression model showed that *NTF3* infusion could modulate the neurotransmitters, such as serotonin and noradrenaline, which are the major targets of currently used antidepressants^33^. *NTF3* was found to be significantly elevated in the temporal region of postmortem brain tissues of patients with depression who were taking antidepressants^34^. Thus, genes within the *KRTAP1-1* co-expression network, including *NTF3*, may be involved in the antidepressant action mechanism.

Previous GWASs using genotyping arrays have reported genetic markers associated with remission or a response following antidepressant treatment^10–14^. However, most of the top SNPs from these studies, including our GWAS study^15^, were not replicated in our WGS results (Supplementary Table S4). Additionally, SNPs that showed significant associations with remission after SSRI treatment in this study were not reported in those previous studies. The main reason for these discrepant results is that our discovery set has a limited sample size to validate previous results that had a sufficient statistical power. In addition, our identified genetic variants (rs3213755) from the *KRTAP1-1* gene were not observed in previous GWASs. In terms of technology, this SNP was not included in either the array-based chip platform or the HapMap3 database used for imputation in a previous meta-analysis of pharmacogenetic studies on the antidepressant response^28^. Therefore, this LOF variant was rarely included in previous studies based on array-chip platforms^10–14^, although rs3213755 is not a rare variant in all populations^35^_._ In addition, a relatively large proportion of elderly patients and differences in ethnicity and antidepressants could be possible factors that caused the lack of agreement between our results and previous GWASs.

Previous pharmacogenetic studies on antidepressant therapy focused on the response rather than remission. The remission always has a lower rate than response, thus studies for remission need more sample size or larger effect size. However, remission is more important outcome than response in depressive patients. A response without remission after antidepressant treatment is associated with a lower degree of functional improvement and higher risk of relapse^5,36^. Our study provides fundamental information that can serve as the basis for a larger WGS study designed to find genetic markers for remission.

This study has several limitations. First, our sample size in the discovery set was insufficient to analyze all the variants identified in the WGS due to high cost^37^. Although we assessed only 100 patients with MDD following SSRI treatment, our study enrolled ethnically homogeneous patients and categorized them appropriately. In addition, our results were validated in an independent replication set, in which the same protocol for patient enrollment was applied. With approximately 10 million independent variants generated using WGS, reaching a genome-wide threshold is very unlikely to identify variants associated with a rare allele frequency, low relative risk, and low fraction of variance explained, even if they are the causal variants^38^. Therefore, using lenient p-value thresholds (p < 0.1 for discovery)^39^, we focused on LOF variants with a high-penetrance conferring moderate or high risk, even if those were common allelic frequency, showing evidence for pathway-related co-expression in human cerebral tissue. Second, there might have been a potential bias related to the physician’s independent choice of antidepressant drug because of the study’s naturalistic design of the study. Additionally, in line with previous GWASs on antidepressant response^10–13^, our study did not include a placebo-treated group. Third, our patients were mostly elderly patients. Thus, the generalizability of our results to patients with depression in other age groups may be limited.

Thus, we observed a significant association between remission to SSRI treatment and a LOF variant, rs3213755, in the *KRTAP1-1* gene in elderly Korean patients with depression. Our functional study using temporal lobe samples is the first step in finding functional evidence between the variant and expression of *KRTAP1-1* in brain tissues. Although functional evidence has been suggested in the animal model systems or biochemical studies, its real function in human brain is not well understood. We believe that *KRTAP1-1* expression in brain tissue may aid in the elucidation of the biological mechanisms of antidepressant drug action and assist in developing precision medicine guidelines for the genomic-based selection of antidepressant treatments. Moreover, additional WGS-based pharmacogenetic studies enrolling various ethnic populations with larger sample sizes are required.

## Methods

### Definition of cohorts

Patients in the discovery set were recruited from the Geropsychiatry Clinic of Samsung Medical Center (*n* = 100, SMC, Seoul, Korea). We enrolled SSRI-treated patients with depression for WGS. All patients were treated with an SSRI (escitalopram). We included 553 additional independent completer patients in the SSRI replication set from the Affective Disorder Clinic of SMC (*n* = 486)^40^ and Korea University Medical Center (*n* = 67; KUMC, Seoul, Korea)^15^. All enrollment processes including inclusion and exclusion criteria, antidepressant treatment, and collecting variables were identical for the replication and discovery sets. Consistent with the current pharmacogenetic strategy related to antidepressants, this study was conducted in naturalistic clinical settings rather than as a placebo-controlled clinical trial^10,11,15^.

### Inclusion and exclusion criteria

The subjects had to meet the following inclusion criteria: (1) age ≥ 18 years, (2) experiencing a current unipolar major depressive episode verified by the *Diagnostic and Statistical Manual of Mental Disorders, Fourth Edition – Text Revision* (DSM-IV-TR) criteria for MDD^40,41^, and (3) be capable of providing informed consent. Diagnosis was based on an initial clinical interview at the clinic, followed by a structured research assessment using the Samsung Psychiatric Evaluation Schedule^15^, which included a semi-structured clinical interview for DSM-IV (SCID)^42^. In accordance with routine clinical procedures, these interviews were individually conducted for each patient. Clinical observations, medical records, past psychiatric histories, and the results of the SCID were assessed by a board-certified psychiatrist before the final diagnosis was made. The minimum score on the 17-item Hamilton scale for depression (HAM-D)^43^ required for enrollment at baseline was 15. The exclusion criteria were (1) pregnancy, significant medical conditions, abnormal laboratory baseline values, or unstable psychiatric features (e.g., suicide attempt in the current episode); (2) a history of substance dependence, seizure, or neurological illness; or (3) concomitant DSM-IV axis I psychiatric disorders (schizophrenia, bipolar affective disorder, primary diagnoses of adjustment disorder, or post-traumatic stress disorder). Patients with MDD who met the DSM-IV criteria for the specifier “severe with psychotic features” were excluded because they were normally taking concurrent antipsychotic medication. None of the patient had received antipsychotics for the current episode before enrollment. Likewise, no patient had received an antidepressant within 2 weeks before enrollment. Additionally, we verified that no patient had received fluoxetine (known to have a long half-life) within the preceding 4 weeks. Most participants overlapped with the participants in our previous GWAS (discovery sample 99/100 and replication sample 415/553)^15^.

### Procedures

Patients received monotherapy for 6 weeks with an antidepressant drug. In the discovery cohort, 100 patients received one SSRI (escitalopram), whereas, in the replication cohort, 553 patients were administered monotherapy with escitalopram, fluoxetine, paroxetine, or sertraline as a permissible SSRI (Table 1). Dose titration was completed within two weeks. Blood samples were withdrawn at the end of the 6th week to estimate plasma drug concentrations. Patients were allowed to take lorazepam (0.5–1 mg) at bedtime for insomnia.

Patients were examined by a psychiatrist who monitored adverse events using the Udvalg for Kliniske Undersogelser (UKU) scale^44^. Symptom severity was evaluated using the HAM-D by a trained rater every 2 weeks. All raters had HAM-D training. The HAM-D and genotype data were not disclosed to the psychiatrist. The rater was blinded to the genotype data. To maintain blinding, a trained research coordinator managed the participants’ data and schedules of participants. At 6 weeks, remission was defined as a HAM-D score < 8. According to standard conventions, a positive response to treatment is defined as a ≥ 50% decrease in the HAM-D score^45,46^.

The replication set also included 67 patients recruited from the Pharmacogenomic Research Center for Psychotropic Drugs of the Department of Psychiatry, KUMC. The procedures followed at this site were very similar to those described for the SMC patients. Detailed protocols are available in previous KUMC reports^47,48^.

### Ethical approval

The protocol was approved by the Ethics Review Board of the SMC and the Ethics Committee of the KUMC, and the procedures were performed according to national ethical guidelines.

### Informed consent

Written informed consent was obtained from all participants.

### WGS analysis and genotyping

WGS data were generated using an Illumina HiSeq X Ten platform. Library construction and sequencing (150-bp paired-end reads) were performed according to the manufacturer’s instructions. The average depth showed 30 × coverage of the whole genome. Sequencing fastq data were aligned with reference genome hg19 containing the decoy sequence using the BWA-mem algorithm of BWA 0.7.10^49^. Duplicate reads were removed using Picard 1.1 (https://broadinstitute.github.io/picard/). Realignment of small INDELs and recalibration of base quality scores were performed using previously known sites (from dbSNP138, Mills and 1000G gold standard INDELs b37 sites, and 1000G phase1 INDELs b37) after removing duplicate reads using GenomeAnalysisTK-3.3-0 (GATK)^50^. Variants were called using HaplotypeCaller in GATK. Variant annotation was conducted using ANNOVAR for RefGene, dbSNP 147, and population frequency from gnomAD^51^. In total, 13,318,214 variants (SNPs and INDELs) were obtained from the raw variant call set after excluding mitochondrial variants. We applied the following hard filter criteria: (1) separating multi-allelic variants, (2) including variants with a total read depth of over 6, (3) including variants with an alternative read depth of over 3, (4) excluding variants with an allelic frequency < 0.1 for SNPs and < 0.2 for INDELs, and (5) excluding variants detected in less than two samples. Loss-Of-Function Transcript Effect Estimator (LOFTEE; https://github.com/konradjk/loftee) was implemented as a variant effect predictor (VEP) to predict high-confidence or low-confidence LOF mutations^52^. In total, 166 LOF mutations were predicted with high confidence using LOFTEE (https://github.com/konradjk/loftee). Overall, we identified 19 variants (7 SNVs and 12 small INDELs) associated with remission. They were annotated as LOF variants with a nominal association *P* value < 0.1. Additionally, we identified 15 variants (3 SNVs and 12 small INDELs) associated with a positive response treatment outcome to antidepressant therapy after applying the same criteria as used for remission. Next, we manually confirmed the existence of variants by capturing relevant regions using an integrative genomics viewer screenshot^53^. The library preparation and clustering methods for WGS were performed as described in the Supplementary Material.

All replication samples were genotyped using TaqMan SNP Genotyping Assays. Detailed descriptions of the genotyping methods for the replication samples are provided in the Supplementary Material.

### Selection of co-expressed genes using an in silico analysis and quantification in brain tissue

To identify functional association networks in public databases, we used the GeneMANIA system (https://genemania.org) to help predict the candidate gene functions^54^. In total, 22 brain tissues acquired from a population of ethnic Korean individuals who had undergone temporal lobectomy for hippocampal sclerosis were used for expression quantification (16 females and 6 males, mean age = 36.5 years [standard deviation = 13.9]). None of the temporal lobectomy specimens showed histological abnormalities. Target gene primers for performing qPCR were designed to quantify the expression of each gene. Detailed descriptions of the in silico analysis and in vitro experiments are provided in the Supplementary Material.

### Statistical analyses

All statistical analyses, including the association analyses, were performed using PLINK, version 1.09 years^55^, and R statistical software (version 3.2.0). We used Fisher’s exact test to calculate the associations between genotypes and treatment outcomes. Continuous variables are presented as the median and interquartile range. Wilcoxon’s rank-sum test was used to compare the continuous variables among groups. Categorical variables are summarized as frequencies and proportions. The chi-square test was used to compare groups with respect to categorical variables. We employed multiple logistic regression to adjust for confounding factors that showed significant differences between the remission and non-remission group or the discovery and replication set. Gene expression correlation analysis was performed using Pearson’s correlation test. Additionally, we employed multiple logistic regression analysis for each significant SNV after adjusting for the possible confounding variables. We examined the reproducibility of previously reported 100 SNPs and 10 SNPs reported by Myung et al*.* (Supplementary Table S4) and Lim et al*.* (Supplementary Table S5), respectively, in the WGS discovery set. We applied nominal threshold of p < 0.05 for quasi-replication and replication of previous genetic studies on the antidepressant response. The direction of effect was estimated by the odds ratio calculated in each study. Statistical significance was defined as *p* < 0.05 or less, with the exception that statistical significance for LOF was defined as a less stringent cut off of *p* < 0.1, because our small sample size could hide true associations trending toward significance. To overcome the low statistical power resulting from the small sample size, more candidate variants were included in the replication set with a threshold *p* < 0.1.

## Supplementary Information

Supplementary Information
