# Supplementary material for: Whole-genome sequencing reveals KRTAP1-1 as a novel genetic variant associated with antidepressant treatment outcomes
Source: Sci Rep. 2021 Feb 25;11:4552. doi: 10.1038/s41598-021-83887-6 (PMC7907209; doi:10.1038/s41598-021-83887-6)
Supplement: Supplementary file 1 — Supplementary Information [file 41598_2021_83887_MOESM1_ESM.docx]

**Supplementary Materials**

**Whole-genome sequencing reveals *KRTAP1-1* as a novel genetic variant associated with antidepressant treatment outcomes**

Jong-Ho Park PhD^1,2*^, Shinn-Won Lim PhD^1*^, Woojae Myung MD, PhD^3*^, Inho Park PhD^4^, Hyeok-Jae Jang MS^1^, Seonwoo Kim PhD^5^, Min-Soo Lee MD, PhD^6^, Hun Soo Chang PhD^7^, DongHo Yum MS^8^, Yeon-Lim Suh MD, PhD^8^, Jong-Won Kim MD, PhD^1,9†^, and Doh Kwan Kim MD, PhD^10†^

^*^These individuals contributed equally to this article as co-first authors.

^†^These individuals contributed equally to this work as co-corresponding authors.

^1^ Department of Health Sciences and Technology, SAIHST, Sungkyunkwan University, Seoul, Korea

^2^ Clinical Genomics Center, Samsung Medical Center, Seoul, Korea

^3^ Department of Neuropsychiatry, Seoul National University Bundang Hospital, Seongnam, Korea

^4^ Precision Medicine Center, Gangnam Severance Hospital, Yonsei University College of Medicine, Seoul, Korea

^5^ Statistics and Data Center, Research Institute for Future Medicine, Samsung Medical Center, Seoul, Korea

^6^ Department of Psychiatry, College of Medicine Korea University, Seoul, Korea

^7^ Soonchunhyang Medical Institute, College of Medicine, Soonchunhyang University, Asan, Korea

^8^ Department of Pathology, Samsung Medical Center, Sungkyunkwan University School of Medicine, Seoul, Korea

^9^ Department of Laboratory Medicine and Genetics, Samsung Medical Center, Sungkyunkwan University School of Medicine, Seoul, Korea

^10^ Department of Psychiatry, Samsung Medical Center, Sungkyunkwan University School of Medicine, Seoul, Korea

**Correspondence:** Drs Doh Kwan Kim and Jong-Won Kim

Doh Kwan Kim, MD, PhD

Department of Psychiatry, Samsung Medical Center, Sungkyunkwan University School of Medicine 81 Irwon-ro, Gangnam-gu, Seoul 135-710, Korea; Tel.: +82-2-3410-3582; Fax: +82-2-3410-0941; E-mail: paulkim@skku.edu

&

Jong-Won Kim, MD, PhD

Department of Laboratory Medicine and Genetics, Samsung Medical Center, Sungkyunkwan University School of Medicine 81 Irwon-ro, Gangnam-gu, Seoul 135-710, Korea; Tel.: +82-2-3410-2709; Fax: +82-2-3410-2719; E-mail: kimjw@skku.edu

**Supplementary Methods**

**Library preparation, clustering, and sequencing.** Libraries were prepared according to the TruSeq nano DNA library prep guide (Illumina). Briefly, 100 ng of quantified genomic DNA was sheared using an LE220 focused ultrasonicator (Covaris, Inc.) with a duty factor of 15%, a peak incident power of 450 W, and 200 cycles per burst for 50 seconds. The sheared DNA fragments were end-repaired and size-selected to obtain DNA fragments with a size of around 350 bp and adenylated according to the manufacturer’s instructions. After ligating indexing adapters to the ends of these DNA fragments, the DNA libraries were enriched using eight cycles of PCR according to the manufacturer’s instructions. The quality and band size of the libraries were assessed using D1000 Screen Tapes (Agilent) on a TapeStation 2200 system (Agilent) after size selection and PCR amplification. The libraries were quantified using a PicoGreen dsDNA quantitation assay (Thermo Fisher Scientific) and estimated using a Victor3 plate reader (PerkinElmer). Illumina utilizes a unique “bridged” amplification reaction that occurs on the surface of the flow cell. A flow cell containing the prepared libraries was prepared using a cBot fluidics station (Illumina). It was subsequently loaded into a HiSeq X-10 sequencer (Illumina) with automated cycles of extension and imaging. The sequencing-by-synthesis cycle was repeated to achieve paired-end reads with a length of 2×150 bp.

**Genotyping for replication samples.** DNA (20 ng/µl) was used to prepare a 384-well reaction plate with a 2X TaqMan Universal PCR Master Mix, 20X Primers, and TaqMan Probes (FAM and VIC) dye mix. We selected seven SNVs (rs1476860, rs3213755, rs139506139, rs877346, rs115678527, rs7021123, and rs1476860) as the candidates associated with antidepressant remission and response in the discovery set. Genotyping was performed on the prepared plate using a real-time PCR system (ABI 7900HT) according to the manufacturer’s instructions. Raw data from the genotyping experiments were analyzed using TaqMan Genotyper Software with the autocalling method for the quality value reflecting the probability of a genotype call.

**Selection of co-expressed genes associated with KRTAP1-1 using an in silico analysis and quantification in human brain tissue.** To identify functional association networks in public databases, we used the GeneMANIA system (https://genemania.org) to help predict the functions of candidate genes^1^. Relevant genes associated with brain and cerebellum tissues were examined using the Database for Annotation, Visualization and Integrated Discovery (DAVID) (https://david.ncifcrf.gov), which provides a comprehensive set of functional annotation tools^2^. Functional annotations associated with drug target and neurological function genes were reviewed using GeneCards (https://www.genecards.org). Finally, protein and RNA expression profiles were obtained from the Human Protein Atlas (https://www.proteinatlas.org/) and GTEx tissue expression database (https://www.gtexportal.org/)^3^. We used the following candidate gene selection criteria: i) genes expressed in the brain, cerebrum, or cerebellum in at least one of the DAVID tissue expression sources, including GNF microarray, CGAP SAGE, CGAP tissue EST, and NCBI Unigene EST; ii) genes for drugs which described in DrugBank, ApexBio, HMDB, or NovoSeek, iii) genes with known neurological functions in RefSeq or UniProt; and iv) genes expressed in the brain tissue in Human Protein Atlas and GTEx. Thus, we finally selected three genes among the 20 genes reviewed from the GeneMANIA database. To determine whether the SNPs identified in the genome-wide association study (GWAS) were functional, variations in the expression level of genes associated with the genotype that generated a knock-out of the said gene were analyzed^4^. In total, 22 brain tissues acquired from a population of ethnic Korean individuals who had undergone brain surgery were used for expression quantification. DNA and total RNA from the paraffin-embedded brain tissues were purified by MagMAX FFPE DNA/RNA Ultra Kit (Applied Biosystems). For qPCR, total RNA was synthesized into cDNA by reverse transcriptase (Applied Biosystems). Target gene primers to perform qPCR were designed to quantify RNA expression of each gene. qPCR amplification was carried out on an ABI 7900 HT Real-Time PCR System (Applied Biosystems). After 45 cycles of 95°C for 5 s and 60°C for 1 min, the dissociation curve analysis for ΔCt was analyzed using SDS 2.4 and ABI RQ manager software. For quantification, the RNA expression of a target gene was normalized against the expression of *GAPDH* as a reference gene (minus ΔCt = *GAPDH* Ct value - TargetGene Ct value).

**Supplementary Results**

We performed gene-based association test using WGS to overcome the insufficient power of a single variant-level approach and to investigate relationships between genes and variants. In the discovery set, we applied SKAT-O method as implemented in the SKAT package^5^ to combine the single variant burden of the genes. To incorporate variants from WGS, the gene region was defined as ± 2bp of a gene. A total of 49,283 sets allocated 628,426 variants with no minor allele frequency threshold (MAF). We identified eight genes, when applying numerous test markers of at least three variants, associated remission following antidepressant treatment at the suggestive threshold of *p* < 0.0001 (Supplementary Table S9). However, further replication studies are required.

**Supplementary References**

1. Warde-Farley, D. *et al.* The GeneMANIA prediction server: biological network integration for gene prioritization and predicting gene function. *Nucleic Acids Res.* **38**, W214-220, <http://doi.org/10.1093/nar/gkq537> (2010).

2. Huang da, W., Sherman, B. T. & Lempicki, R. A. Bioinformatics enrichment tools: paths toward the comprehensive functional analysis of large gene lists. *Nucleic Acids Res.* **37**, 1-13, <http://doi.org/10.1093/nar/gkn923> (2009).

3. GTEx Consortium. The Genotype-Tissue Expression (GTEx) project. *Nat. Genet.* **45**, 580-585, <http://doi.org/10.1038/ng.2653> (2013).

4. Westra, H. J. *et al.* Systematic identification of trans eQTLs as putative drivers of known disease associations. *Nat. Genet.* **45**, 1238-1243, <http://doi.org/10.1038/ng.2756> (2013).

5. Lee, S. *et al.* Optimal unified approach for rare-variant association testing with application to small-sample case-control whole-exome sequencing studies. *Am. J. Hum. Genet.* **91**, 224-237, <http://doi.org/10.1016/j.ajhg.2012.06.007> (2012).

A.


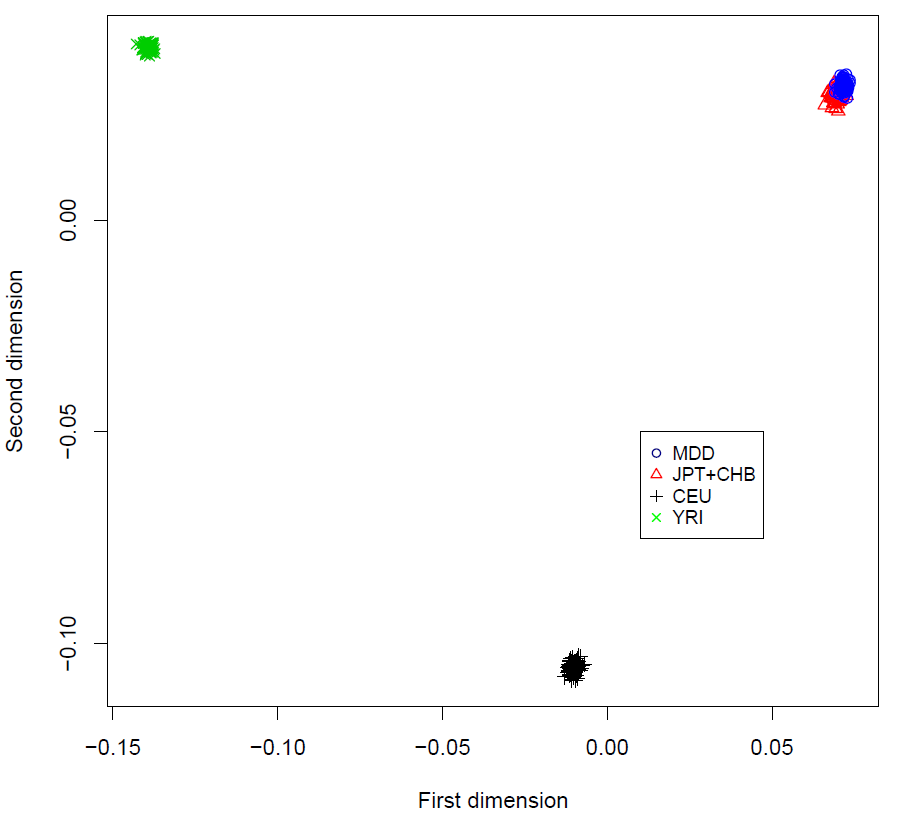


B.


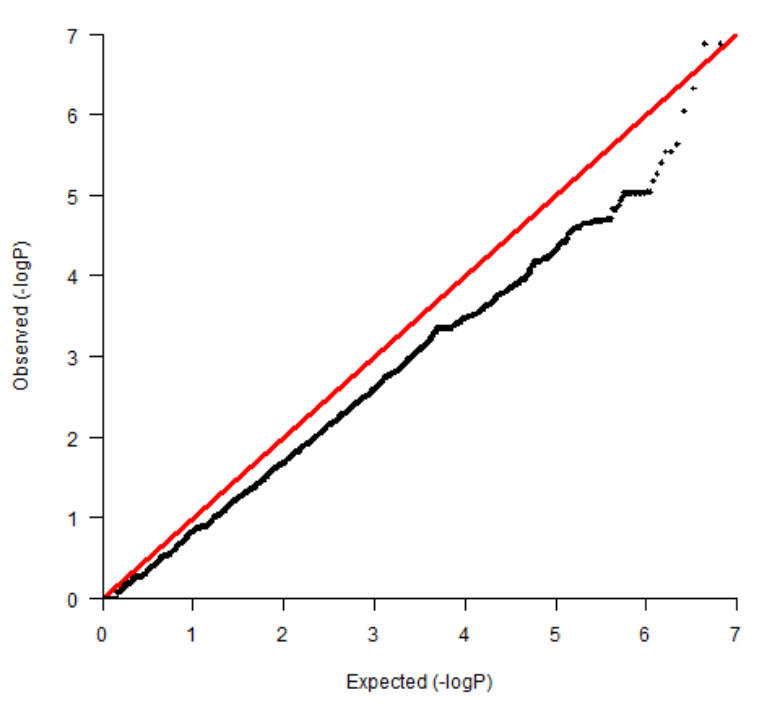


**Supplementary Fig. 1 Multi-dimensional scaling and Quantile-quantile (QQ) plot of 100 patients with MDD in the discovery set. A**. Multi-dimensional scaling analysis revealed that there was no population stratification of the 100 patients with MDD used in the discovery set. Multi-dimensional scaling analysis was estimated for 270 individuals in the HapMap Project data. We used 90 individuals from the JPT + CHB population (Japanese in Tokyo and Han Chinese in Beijing), 90 from the CEU population (Utah Residents (CEPH) with Northern and Western European ancestry), and 90 from the YRI population (Yoruba in Ibadan, Nigeria). **B**. We have estimated genomic inflation factor based on median chisq λ = 1 and identified quantile-quantile plot (QQ plot) from the distribution of expected p value and observed p value for all variants from WGS.


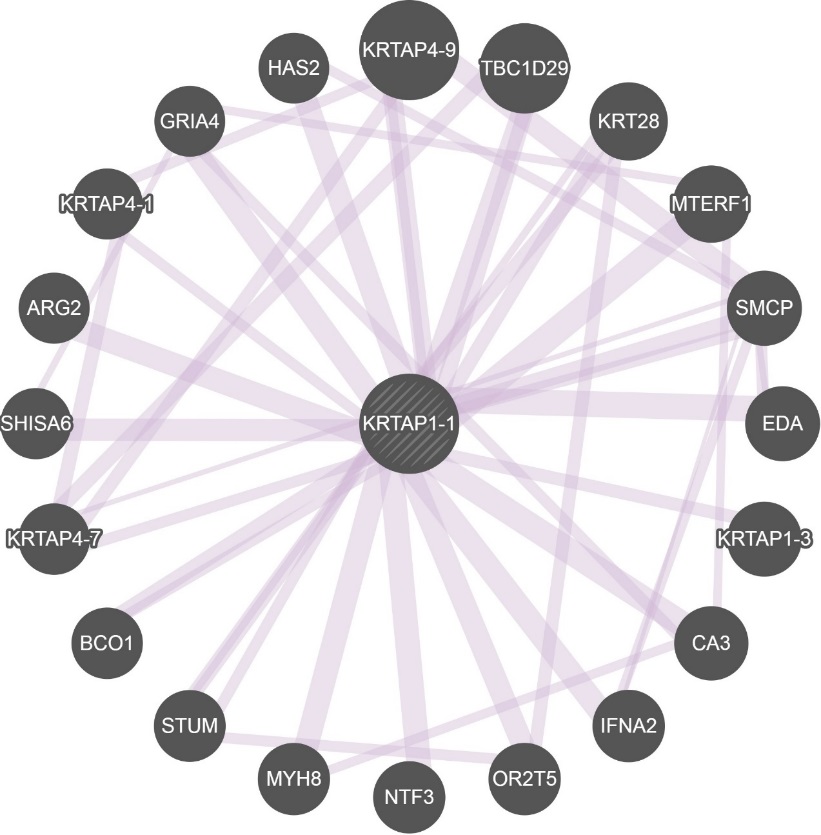


**Supplementary Fig. 2 Co-expressed network from GeneMANIA.** Genes co-expressed with *KRTAP1-1* from the GeneMANIA database are displayed. Links of expression with each gene were collected from the Gene Expression Omnibus (GEO).

**
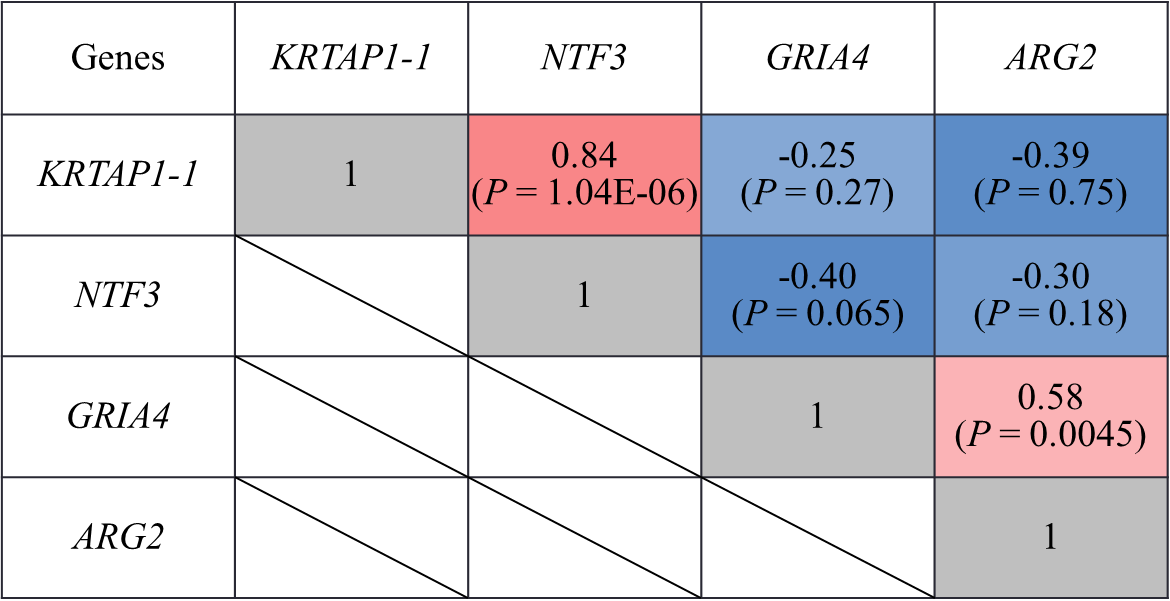
**

**Supplementary Fig. 3. Correlation results of the quantified expression values for *KRTAP1-1* and the co-expressed genes in brain tissues.** Correlation coefficients and *p* values among the two genes were calculated using Pearson’s product-moment correlation.

**Supplementary Table S1 List of variants (*n* = 18) associated with remission following antidepressant treatment in the discovery set (*p* < 1.00E-05).**

| **Chromosome** | **SNP** | **Position^†^** | **Gene annotation** | **Assigned genes** | **Minor/major**  **allele** | **MAF in**  **non-remitted** | **MAF in**  **remitted** | ***p* value^‡^** | **OR (CI, 95%)^‡^** |
| --- | --- | --- | --- | --- | --- | --- | --- | --- | --- |
| 1 | rs723238 | 164663449 | intronic | *PBX1* | G/A | 0.63 | 0.35 | 9.79E-06 | 15.55 (3.43–70.38) |
| 1 | rs2171692 | 164666940 | intronic | *PBX1* | A/G | 0.63 | 0.35 | 9.79E-06 | 15.55 (3.43–70.38) |
| 1 | rs12143203 | 164667597 | intronic | *PBX1* | T/C | 0.63 | 0.35 | 9.79E-06 | 15.55 (3.43–70.38) |
| 1 | rs10918058 | 164670322 | intronic | *PBX1* | G/A | 0.64 | 0.37 | 9.79E-06 | 15.55 (3.43–70.38) |
| 1 | rs10918059 | 164670350 | intronic | *PBX1* | C/T | 0.64 | 0.37 | 9.79E-06 | 15.55 (3.43–70.38) |
| 1 | rs10800045 | 164671417 | intronic | *PBX1* | A/C | 0.68 | 0.38 | 9.79E-06 | 15.55 (3.43–70.38) |
| 1 | rs12760919 | 164675868 | intronic | *PBX1* | T/C | 0.68 | 0.38 | 9.79E-06 | 15.55 (3.43–70.38) |
| 2 | rs76946251 | 57818747 | intergenic | *CCDC85A,VRK2* | G/A | 0.26 | 0.02 | 1.26E-06 | 21.64 (4.61–101.53) |
| 3 | rs2873447 | 100659095 | intronic | *ABI3BP* | C/T | 0.11 | 0.40 | 2.11E-06 | 0.12 (0.045–0.30) |
| 3 | rs144799752 | 100674334 | intronic | *ABI3BP* | C/CTTAT | 0.18 | 0.48 | 4.49E-06 | 0.13 (0.053–0.32) |
| 3 | rs57335546 | 100688476 | intronic | *ABI3BP* | A/C | 0.11 | 0.39 | 5.18E-06 | 0.13 (0.049–0.32) |
| 3 | rs66827303 | 100700716 | intronic | *ABI3BP* | A/G | 0.16 | 0.47 | 4.03E-06 | 0.13 (0.051–0.31) |
| 4 | rs112968747 | 49181808 | intergenic | *CWH43,NONE* | C/G | 0.39 | 0.14 | 1.68E-06 | 9.91 (3.67–26.76) |
| 13 | rs9544610 | 78398594 | ncRNA_intronic | *EDNRB-AS1* | A/G | 0.34 | 0.09 | 4.11E-06 | 8.33 (3.28–21.19) |
| 13 | rs1041619 | 78409150 | ncRNA_intronic | *EDNRB-AS1* | T/A | 0.36 | 0.11 | 7.76E-06 | 7.43 (3.00–18.40) |
| 13 | rs9574113 | 78418131 | ncRNA_intronic | *EDNRB-AS1* | G/A | 0.38 | 0.11 | 2.67E-06 | 8.31 (3.33–20.75) |
| 14 | rs75309282 | 56380629 | intergenic | *LINC00520,PELI2* | T/C | 0.01 | 0.22 | 4.18E-06 | 0.04 (0.0046–0.28) |
| 21 | 21:9842673^a^ | 9842673 | intergenic | *MIR3687-1,TEKT4P2* | C/G | 0.22 | 0.02 | 3.48E-06 | 20.40 (4.29–96.92) |

Abbreviations: MAF, minor allele frequency; OR, odds ratio; CI, confidence interval; SNP, single nucleotide polymorphism

^†^Physical position based on human reference genome build hg 19 (GRCh37).

^‡^*p* value and OR were calculated using a dominant disease model.

^a^In the case of a non-existent rsID, the SNP was presented to the chromosome and genomic position as “chromosome:position.”

**Supplementary Table S2 List of variants (*n* = 18) associated with a response to antidepressant treatment in the discovery set (*p* < 1.00E-05).**

| **Chromosome** | **SNP** | **Position^†^** | **Gene annotation** | **Assigned genes** | **Minor/major**  **allele** | **MAF in**  **non-response** | **MAF in**  **response** | ***P* value^‡^** | **OR (CI, 95%)^‡^** |
| --- | --- | --- | --- | --- | --- | --- | --- | --- | --- |
| 2 | rs76706117 | 143475367 | intergenic | *LRP1B,KYNU* | C/T | 0.09 | 0.35 | 4.70E-07 | 0.093 (0.035–0.24) |
| 2 | rs60412085 | 143517762 | intergenic | *LRP1B,KYNU* | C/T | 0.07 | 0.35 | 6.29E-08 | 0.073 (0.026–0.20) |
| 2 | rs58147340 | 143536066 | intergenic | *LRP1B,KYNU* | T/TA | 0.08 | 0.35 | 1.33E-07 | 0.073 (0.026–0.20) |
| 2 | rs1472079 | 143541212 | intergenic | *LRP1B,KYNU* | T/G | 0.08 | 0.35 | 1.33E-07 | 0.073 (0.026–0.20) |
| 2 | rs6712291 | 143550868 | intergenic | *LRP1B,KYNU* | C/T | 0.10 | 0.36 | 9.05E-07 | 0.10 (0.040–0.26) |
| 6 | rs9390612 | 149120342 | intronic | *UST* | A/G | 0.21 | 0.43 | 9.12E-06 | 0.12 (0.044–0.33) |
| 14 | rs71412080 | 22751798 | intergenic | *OR4E2,DAD1* | A/G | 0.05 | 0.28 | 9.34E-06 | 0.11 (0.040–0.31) |
| 14 | rs2001020 | 22752095 | intergenic | *OR4E2,DAD1* | G/C | 0.05 | 0.28 | 9.34E-06 | 0.11 (0.040–0.31) |
| 14 | rs71412081 | 22753178 | intergenic | *OR4E2,DAD1* | A/G | 0.05 | 0.28 | 9.34E-06 | 0.11 (0.040–0.31) |
| 14 | rs74242648 | 22753793 | intergenic | *OR4E2,DAD1* | C/T | 0.05 | 0.28 | 9.34E-06 | 0.11 (0.040–0.31) |
| 14 | rs2001021 | 22757152 | intergenic | *OR4E2,DAD1* | C/T | 0.05 | 0.28 | 9.34E-06 | 0.11 (0.040–0.31) |
| 14 | rs2001022 | 22757266 | intergenic | *OR4E2,DAD1* | T/C | 0.05 | 0.28 | 9.34E-06 | 0.11 (0.040–0.31) |
| 14 | rs12895921 | 22759249 | intergenic | *OR4E2,DAD1* | C/T | 0.05 | 0.28 | 9.34E-06 | 0.11 (0.040–0.31) |
| 14 | rs74654226 | 22760708 | intergenic | *OR4E2,DAD1* | A/G | 0.05 | 0.28 | 9.34E-06 | 0.11 (0.040–0.31) |
| 14 | rs17793685 | 22761800 | intergenic | *OR4E2,DAD1* | G/C | 0.05 | 0.28 | 9.34E-06 | 0.11 (0.040–0.31) |
| 15 | rs11855728 | 89525997 | intergenic | *MFGE8,ABHD2* | G/T | 0.30 | 0.04 | 9.29E-06 | 11.71 (3.26–42.10) |
| 16 | rs76978596 | 33513668 | ncRNA_intronic | *RNU6-76P* | T/G | 0.31 | 0.51 | 3.91E-06 | 0.022 (0.0013–0.38) |
| 17 | 17:56659927^a^ | 56659927 | intronic | *TEX14* | C/CT | 0.01 | 0.20 | 6.60E-06 | 0.028 (0.0034–0.23) |

Abbreviations: MAF, minor allele frequency; OR, odds ratio; CI, confidence interval; SNP, single nucleotide polymorphism

^†^Physical position based on human reference genome build hg 19 (GRCh37).

^‡^*p* value and OR were calculated using a dominant disease model.

^a^In the case of a non-existent rsID, the SNP was presented to the chromosome and genomic position as “chromosome:position.”**Supplementary Table S3 Loss-of-function candidate variants associated with remission after only escitalopram treatment in patients with MDD (discovery set, *n* = 100; replication set, *n* = 193).**

| **Chromosome** | **SNP** | **Position^†^** | **Gene** | **Minor /**  **major allele** | **Cohort**^a^ | **MAF**  **in non-remitted** | **MAF**  **in remitted** | ***p* value^‡^** | **OR (CI, 95%)^‡^** |
| --- | --- | --- | --- | --- | --- | --- | --- | --- | --- |
| 9 | rs1476860 | 125391241 | *OR1B1* | A/G | Discovery | 0.391 | 0.333 | 0.077^*^ | 4.33 (0.92–20.43)^*^ |
|  |  |  |  |  | Replication | 0.383 | 0.375 | 0.830^*^ | 1.15 (0.48–2.76)^*^ |
|  |  |  |  |  | Combined | 0.386 | 0.359 | 0.166^*^ | 1.73 (0.82–3.66)^*^ |
| 17 | rs3213755 | 39197499 | *KRTAP1-1* | A/G | Discovery | 0.234 | 0.125 | 0.018 | 3.09 (1.22–7.80) |
|  |  |  |  |  | Replication | 0.401 | 0.218 | 0.006 | 2.50 (1.31–4.77) |
|  |  |  |  |  | Combined | 0.348 | 0.181 | 0.00014 | 2.75 (1.63–4.65) |
| 19 | rs139506139 | 44117804 | *SRRM5* | T/C | Discovery | 0 | 0.042 | 0.044 | 0.07 (0.0037-1.48) |
|  |  |  |  |  | Replication | 0.004 | 0.036 | 0.026 | 0.096 (0.010–0.88) |
|  |  |  |  |  | Combined | 0.002 | 0.038 | 0.002 | 0.061 (0.0074–0.50) |
| 21 | rs877346 | 31744127 | *KRTAP13-2* | T/A | Discovery | 0.266 | 0.125 | 0.055 | 2.49 (1.01–6.12) |
|  |  |  |  |  | Replication | 0.210 | 0.109 | 0.042 | 2.22 (1.07–4.59) |
|  |  |  |  |  | Combined | 0.228 | 0.115 | 0.004 | 2.27 (1.29–3.99) |

Abbreviations: MAF, minor allele frequency; OR, odds ratio; CI, confidence interval; SNP, single nucleotide polymorphism; ESCI, escitalopram; NA, not available

^†^Physical position based on human reference genome build hg 19 (GRCh37).

^‡^*p* value and OR were calculated using a dominant disease model.

^*^*p* value was calculated using a recessive disease model.

^a^In the discovery set, remitted, *n* = 36, non-remitted, *n* = 64; in the replication set, remitted, *n* = 56, non-remitted, *n* = 137.**Supplementary Table S4. Replication of the top 100 SNPs that were reported in our previous GWAS for antidepressant response in Korean population (Myung et al.).**

| **The top 100 SNPs that were reported in previous GWAS for antidepressant response (Myung *et al* .)** | | | | | | **Quasi-replication for remission in our WGS data** | | | **Replication for response in our WGS data** | |
| --- | --- | --- | --- | --- | --- | --- | --- | --- | --- | --- |
| **Rank** | **SNP** | **Chromosome** | **Position^†^** | ***P* value** | **OR (95% CI)** | ***P*** | **value^‡^ OR (CI 95%)^‡^** | **Consistency of the direction** | ***P* value^‡^ OR (CI 95%)^‡^** | **Consistency of the direction** |
| 1 | rs12698828 | 7 | 69446594 | 3.28E-06 | 3.53 (1.99-6.26) | 0.563 0.71 (0.23-2.25) | | Inconsistent | 0.558 1.61 (0.52-4.99) | Consistent |
| 2 | rs7785360 | 7 | 69409378 | 2.00E-08 | 3.53 (1.99-6.26) | 0.563 0.71 (0.23-2.25) | | Inconsistent | 0.558 1.61 (0.52-4.99) | Consistent |
| 3 | rs10924309 | 1 | 245863222 | 6.00E-06 | 0.55 (0.42-0.71) | 0.014 0.27 (0.1-0.75) | | Consistent | 0.129 0.49 (0.21-1.15) | Consistent |
| 4 | rs8017553 | 14 | 23737522 | 9.45E-06 | 0.50 (0.36-0.69) | 0.294 0.63 (0.27-1.44) | | Consistent | 0.835 0.84 (0.37-1.91) | Consistent |
| 5 | rs3811180 | 14 | 23733725 | 1.13E-05 | 0.50 (0.36-0.70) | 0.294 0.63 (0.27-1.44) | | Consistent | 0.835 0.84 (0.37-1.91) | Consistent |
| 6 | rs9635953 | 18 | 47345929 | 1.61E-05 | 0.56 (0.42-0.73) | 0.831 0.89 (0.38-2.11) | | Consistent | 0.402 0.7 (0.3-1.6) | Consistent |
| 7 | rs16912747 | 12 | 17554177 | 1.63E-05 | 0.46 (0.32-0.67) | 0.175 0.52 (0.22-1.26) | | Consistent | 0.265 0.54 (0.22-1.34) | Consistent |
| 8 | rs10515209 | 5 | 75128183 | 1.71E-05 | 2.56 (1.67-3.92) | 0.079 2.89 (0.89-9.4) | | Consistent | 0.026 3.13 (1.16-8.47) | Consistent |
| 9 | rs948667 | 18 | 47368230 | 1.85E-05 | 0.56 (0.42-0.74) | 0.829 0.83 (0.35-1.97) | | Consistent | 0.294 0.63 (0.27-1.44) | Consistent |
| 10 | rs11752615 | 6 | 84380660 | 1.90E-05 | 1.97 (1.45-2.67) | 0.663 1.27 (0.53-3.05) | | Consistent | 0.031 2.71 (1.16-6.37) | Consistent |
| 11 | rs1334328 | 6 | 97094444 | 1.98E-05 | 0.55 (0.42-0.72) | 0.297 1.63 (0.72-3.72) | | Inconsistent | 0.688 0.85 (0.38-1.89) | Consistent |
| 12 | rs2091104 | 7 | 145180205 | 2.27E-05 | 0.55 (0.41-0.72) | 0.012 0.31 (0.13-0.77) | | Consistent | 0.040 0.41 (0.18-0.93) | Consistent |
| 13 | rs7244563 | 18 | 47290365 | 2.50E-05 | 0.56 (0.43-0.74) | 0.663 0.78 (0.33-1.88) | | Consistent | 0.389 0.64 (0.28-1.49) | Consistent |
| 14 | rs4332094 | 8 | 128856908 | 2.79E-05 | 1.92 (1.42-2.60) | 0.275 0.61 (0.26-1.46) | | Inconsistent | 0.385 1.52 (0.65-3.56) | Consistent |
| 15 | rs10985450 | 9 | 124685381 | 3.14E-05 | 1.76 (1.34-2.31) | 0.406 1.51 (0.66-3.43) | | Consistent | 0.841 1.11 (0.5-2.46) | Consistent |
| 16 | rs877836 | 19 | 29762562 | 3.29E-05 | 1.75 (1.34-2.27) | 1.000 1.01 (0.43-2.36) | | Consistent | 0.396 1.56 (0.66-3.64) | Consistent |
| 17 | rs2276170 | 18 | 47369758 | 3.81E-05 | 0.57 (0.43-0.75) | 0.668 0.78 (0.33-1.84) | | Consistent | 0.208 0.57 (0.25-1.3) | Consistent |
| 18 | rs10924305 | 1 | 245856763 | 4.09E-05 | 0.58 (0.45-0.76) | 0.009 0.27 (0.1-0.71) | | Consistent | 0.208 0.57 (0.25-1.30) | Consistent |
| 19 | rs12353109 | 9 | 32282392 | 4.21E-05 | 2.15 (1.48-3.13) | 0.269 1.8 (0.73-4.46) | | Consistent | 0.129 2.03 (0.87-4.75) | Consistent |
| 20 | rs1520313 | 4 | 12782817 | 4.64E-05 | 0.59 (0.46-0.77) | 0.651 0.79 (0.31-1.99) | | Consistent | 0.112 0.46 (0.19-1.12) | Consistent |
| 21 | rs2165872 | 1 | 239826988 | 4.98E-05 | 0.51 (0.37-0.71) | 0.213 1.78 (0.78-4.09) | | Inconsistent | 0.839 1.14 (0.51-2.55) | Inconsistent |
| 22 | rs11754643 | 6 | 84313180 | 5.14E-05 | 1.88 (1.39-2.55) | 0.508 1.46 (0.6-3.55) | | Consistent | 0.017 2.97 (1.26-7.04) | Consistent |
| 23 | rs10772898 | 12 | 16329053 | 5.17E-05 | 0.58 (0.44-0.76) | 0.390 0.64 (0.27-1.53) | | Consistent | 0.011 0.33 (0.14-0.77) | Consistent |
| 24 | rs1867264 | 1 | 239845277 | 5.38E-05 | 0.51 (0.37-0.70) | 0.213 1.78 (0.78-4.09) | | Inconsistent | 0.839 1.14 (0.51-2.55) | Inconsistent |
| 25 | rs1867265 | 1 | 239840107 | 5.38E-05 | 0.51 (0.37-0.70) | 0.213 1.78 (0.78-4.09) | | Inconsistent | 0.839 1.14 (0.51-2.55) | Inconsistent |
| 26 | rs11669149 | 19 | 29765154 | 5.46E-05 | 2.06 (1.43-2.97) | 0.017 3.91 (1.22-12.5) | | Consistent | 0.098 2.4 (0.95-6.03) | Consistent |
| 27 | rs17366799 | 4 | 12778849 | 5.83E-05 | 0.59 (0.46-0.77) | 1.000 0.9 (0.37-2.17) | | Consistent | 0.192 0.55 (0.23-1.28) | Consistent |
| 28 | rs12669573 | 7 | 145193978 | 5.83E-05 | 0.59 (0.45-0.76) | 0.055 0.4 (0.16-0.99) | | Consistent | 0.142 0.51 (0.22-1.17) | Consistent |
| 29 | rs3826263 | 16 | 23610658 | 5.86E-05 | 3.18 (1.78-5.67) | 0.079 3.92 (0.83-18.63) | | Consistent | 0.075 3.19 (0.98-10.38) | Consistent |
| 30 | rs6700721 | 1 | 245854173 | 5.91E-05 | 0.59 (0.45-0.76) | 0.005 0.26 (0.1-0.67) | | Consistent | 0.142 0.51 (0.22-1.17) | Consistent |
| 31 | rs1104703 | NA | NA | 6.11E-05 | 1.66 (1.28-2.14) | NA NA | | NA | NA NA | NA |
| 32 | rs6453086 | 5 | 74107614 | 6.77E-05 | 2.02 (1.42-2.88) | 0.258 1.83 (0.72-4.69) | | Consistent | 0.044 2.67 (1.11-6.43) | Consistent |
| 33 | rs984230 | 23 | 93564580 | 6.81E-05 | 0.55 (0.41-0.72) | 0.288 0.6 (0.25-1.43) | | Consistent | 0.021 0.36 (0.15-0.83) | Consistent |
| 34 | rs12698891 | 7 | 69765505 | 7.19E-05 | 3.13 (1.73-5.69) | 0.196 0.42 (0.12-1.5) | | Inconsistent | 1.000 0.84 (0.23-3.08) | Inconsistent |
| 35 | rs2297988 | 10 | 99118382 | 7.28E-05 | 1.77 (1.33-2.36) | 0.211 1.76 (0.75-4.13) | | Consistent | 0.040 2.44 (1.07-5.56) | Consistent |
| 36 | rs2044410 | 5 | 74148251 | 7.37E-05 | 2.00 (1.41-2.85) | 0.258 1.83 (0.72-4.69) | | Consistent | 0.044 2.67 (1.11-6.43) | Consistent |
| 37 | rs2114213 | 1 | 72404727 | 7.43E-05 | 0.59 (0.46-0.77) | 0.182 0.51 (0.2-1.3) | | Consistent | 0.127 0.50 (0.21-1.18) | Consistent |
| 38 | rs12698811 | 7 | 69348127 | 7.61E-05 | 3.04 (1.70-5.44) | 0.248 0.51 (0.16-1.59) | | Inconsistent | 1.000 1.15 (0.37-3.6) | Consistent |
| 39 | rs1402038 | 4 | 12791179 | 7.62E-05 | 0.61 (0.47-0.78) | 0.810 0.79 (0.3-2.07) | | Consistent | 0.357 0.65 (0.26-1.61) | Consistent |
| 40 | rs3741255 | 11 | 61516910 | 7.87E-05 | 0.47 (0.32-0.69) | 0.626 0.73 (0.28-1.86) | | Consistent | 0.484 0.69 (0.26-1.8) | Consistent |
| 41 | rs750123 | 18 | 73627838 | 7.98E-05 | 0.55 (0.41-0.74) | 0.527 0.72 (0.31-1.63) | | Consistent | 0.063 0.43 (0.18-1) | Consistent |
| 42 | rs6701023 | 1 | 245854503 | 8.34E-05 | 0.60 (0.46-0.77) | 0.009 0.27 (0.1-0.71) | | Consistent | 0.208 0.57 (0.25-1.30) | Consistent |
| 43 | rs4785465 | 16 | 50922594 | 9.34E-05 | 0.60 (0.46-0.78) | 0.390 0.64 (0.27-1.53) | | Consistent | 0.093 0.47 (0.21-1.09) | Consistent |
| 44 | rs2263942 | 12 | 82420006 | 9.37E-05 | 1.82 (1.35-2.46) | 0.400 1.56 (0.67-3.61) | | Consistent | 0.538 1.36 (0.61-3.04) | Consistent |
| 45 | rs7244073 | 18 | 47360819 | 9.37E-05 | 0.58 (0.44-0.77) | 0.403 0.69 (0.29-1.61) | | Consistent | 0.403 0.66 (0.29-1.49) | Consistent |
| 46 | rs7244102 | 18 | 47360874 | 1.03E-04 | 0.58 (0.44-0.77) | 0.403 0.69 (0.29-1.61) | | Consistent | 0.403 0.66 (0.29-1.49) | Consistent |
| 47 | rs6610384 | 23 | 39934488 | 1.05E-04 | 0.60 (0.44-0.82) | 0.831 1.12 (0.47-2.65) | | Inconsistent | 1.000 1 (0.43-2.31) | Inconsistent |
| 48 | rs11252385 | 10 | 4217047 | 1.07E-04 | 0.31 (0.17-0.57) | 0.563 0.71 (0.23-2.25) | | Consistent | 0.041 0.21 (0.04-1) | Consistent |
| 49 | rs10123547 | 9 | 32278058 | 1.07E-04 | 2.06 (1.41-3.01) | 0.126 2.1 (0.82-5.35) | | Consistent | 0.082 2.22 (0.94-5.22) | Consistent |
| 50 | rs1867263 | 1 | 239807920 | 1.09E-04 | 0.53 (0.39-0.73) | 0.213 1.78 (0.78-4.09) | | Inconsistent | 0.839 1.14 (0.51-2.55) | Inconsistent |
| 51 | rs1993370 | 5 | 74159137 | 1.10E-04 | 1.97 (1.38-2.80) | 0.258 1.83 (0.72-4.69) | | Consistent | 0.044 2.67 (1.11-6.43) | Consistent |
| 52 | rs6136530 | 20 | 18810705 | 1.22E-04 | 0.59 (0.45-0.78) | 0.671 0.77 (0.33-1.8) | | Consistent | 0.683 0.84 (0.37-1.9) | Consistent |
| 53 | rs6136525 | 20 | 18806046 | 1.29E-04 | 0.59 (0.45-0.78) | 0.671 0.77 (0.33-1.8) | | Consistent | 0.683 0.84 (0.37-1.9) | Consistent |
| 54 | rs10882907 | 10 | 99125788 | 1.29E-04 | 1.74 (1.31-2.32) | 0.211 1.76 (0.75-4.13) | | Consistent | 0.040 2.44 (1.07-5.56) | Consistent |
| 55 | rs13212099 | 6 | 84300181 | 1.41E-04 | 1.74 (1.31-2.30) | 0.144 0.51 (0.22-1.17) | | Inconsistent | 0.228 1.66 (0.74-3.72) | Consistent |
| 56 | rs1030100 | 19 | 29357498 | 1.42E-04 | 1.70 (1.30-2.22) | 0.535 1.35 (0.59-3.06) | | Consistent | 0.685 1.22 (0.55-2.73) | Consistent |
| 57 | rs10970922 | 9 | 32331376 | 1.42E-04 | 2.17 (1.44-3.25) | 0.359 1.71 (0.67-4.39) | | Consistent | 0.177 1.97 (0.82-4.73) | Consistent |
| 58 | rs676592 | 4 | 82733530 | 1.42E-04 | 0.62 (0.48-0.80) | 0.821 1.12 (0.46-2.75) | | Inconsistent | 0.653 0.76 (0.31-1.81) | Consistent |
| 59 | rs10062244 | 5 | 74169045 | 1.42E-04 | 2.03 (1.39-2.96) | 0.168 2.17 (0.82-5.74) | | Consistent | 0.024 2.96 (1.21-7.20) | Consistent |
| 60 | rs10491377 | 5 | 36707683 | 1.47E-04 | 0.52 (0.37-0.73) | 0.394 0.68 (0.29-1.59) | | Consistent | 0.396 0.64 (0.27-1.51) | Consistent |
| 61 | rs12046366 | 1 | 189022599 | 1.48E-04 | 0.61 (0.47-0.79) | 0.055 0.4 (0.16-0.99) | | Consistent | 0.021 0.36 (0.15-0.83) | Consistent |
| 62 | rs7555408 | 1 | 189022973 | 1.48E-04 | 0.61 (0.47-0.79) | 0.055 0.4 (0.16-0.99) | | Consistent | 0.021 0.36 (0.15-0.83) | Consistent |
| 63 | rs2328452 | 20 | 19765174 | 1.52E-04 | 1.87 (1.35-2.58) | 0.045 2.83 (1.08-7.44) | | Consistent | 0.00020 5.44 (2.21-13.43) | Consistent |
| 64 | rs6747145 | 2 | 73609177 | 1.54E-04 | 1.73 (1.29-2.32) | 0.835 0.84 (0.37-1.9) | | Inconsistent | 0.541 1.40 (0.63-3.12) | Consistent |
| 65 | rs7576824 | 2 | 73611281 | 1.54E-04 | 1.73 (1.29-2.32) | 0.835 0.84 (0.37-1.9) | | Inconsistent | 0.541 1.40 (0.63-3.12) | Consistent |
| 66 | rs1024881 | 19 | 29356445 | 1.54E-04 | 1.70 (1.30-2.22) | 0.535 1.35 (0.59-3.06) | | Consistent | 0.685 1.22 (0.55-2.73) | Consistent |
| 67 | rs11663529 | 18 | 8780073 | 1.55E-04 | 0.60 (0.46-0.78) | 0.658 0.79 (0.32-1.93) | | Consistent | 0.514 0.73 (0.31-1.73) | Consistent |
| 68 | rs3800544 | 6 | 170058374 | 1.56E-04 | 2.22 (1.49-3.32) | 0.793 1.27 (0.44-3.71) | | Consistent | 0.604 1.45 (0.53-3.97) | Consistent |
| 69 | rs17738645 | 5 | 74158340 | 1.63E-04 | 2.01 (1.38-2.93) | 0.168 2.17 (0.82-5.74) | | Consistent | 0.024 2.96 (1.21-7.20) | Consistent |
| 70 | rs9564791 | 13 | 71798043 | 1.65E-04 | 1.67 (1.29-2.16) | 0.833 1.17 (0.51-2.67) | | Consistent | 0.837 1.15 (0.51-2.59) | Consistent |
| 71 | rs6495009 | 15 | 33575924 | 1.68E-04 | 0.61 (0.47-0.79) | 0.012 0.25 (0.09-0.73) | | Consistent | 0.001 0.2 (0.08-0.5) | Consistent |
| 72 | rs7173234 | 15 | 33576883 | 1.68E-04 | 0.61 (0.47-0.79) | 0.012 0.25 (0.09-0.73) | | Consistent | 0.001 0.2 (0.08-0.5) | Consistent |
| 73 | rs11756746 | 6 | 84286477 | 1.74E-04 | 1.74 (1.31-2.30) | 0.144 0.51 (0.22-1.17) | | Inconsistent | 0.228 1.66 (0.74-3.72) | Consistent |
| 74 | rs6818398 | 4 | 153943571 | 1.79E-04 | 0.54 (0.40-0.74) | 0.676 1.21 (0.52-2.81) | | Inconsistent | 0.537 0.75 (0.33-1.73) | Consistent |
| 75 | rs12519432 | 5 | 9699758 | 1.80E-04 | 0.55 (0.41-0.75) | 0.292 0.6 (0.26-1.37) | | Consistent | 0.303 0.62 (0.27-1.40) | Consistent |
| 76 | rs1912911 | 1 | 189047200 | 1.85E-04 | 0.62 (0.48-0.80) | 0.055 0.4 (0.16-0.99) | | Consistent | 0.021 0.36 (0.15-0.83) | Consistent |
| 77 | rs4388707 | 1 | 189034234 | 1.91E-04 | 0.61 (0.46-0.79) | 0.007 0.3 (0.12-0.72) | | Consistent | 0.008 0.32 (0.14-0.74) | Consistent |
| 78 | rs4700160 | 5 | 66002788 | 1.91E-04 | 0.61 (0.47-0.80) | 0.065 0.36 (0.13-0.98) | | Consistent | 0.177 0.51 (0.21-1.22) | Consistent |

| 79 | rs4635018 | 10 | 99137839 | 1.92E-04 | 1.71 (1.28-2.28) | 0.292 1.66 (0.71-3.88) | Consistent | 0.065 2.21 (0.97-5.02) | Consistent |
| --- | --- | --- | --- | --- | --- | --- | --- | --- | --- |
| 80 | rs1003404 | 7 | 69054960 | 1.93E-04 | 2.80 (1.60-4.90) | 0.258 0.5 (0.17-1.47) | Inconsistent | 1.000 0.88 (0.29-2.66) | Inconsistent |
| 81 | rs2861430 | 1 | 189032480 | 1.93E-04 | 0.62 (0.48-0.80) | 0.055 0.4 (0.16-0.99) | Consistent | 0.021 0.36 (0.15-0.83) | Consistent |
| 82 | rs6428096 | 1 | 189010340 | 1.93E-04 | 0.62 (0.48-0.80) | 0.055 0.4 (0.16-0.99) | Consistent | 0.021 0.36 (0.15-0.83) | Consistent |
| 83 | rs7947523 | 11 | 80932827 | 1.98E-04 | 0.51 (0.35-0.74) | 0.817 0.82 (0.33-2.02) | Consistent | 0.176 0.5 (0.19-1.28) | Consistent |
| 84 | rs17205838 | 16 | 16140041 | 2.09E-04 | 2.17 (1.43-3.28) | 0.306 1.9 (0.63-5.75) | Consistent | 0.320 1.67 (0.62-4.47) | Consistent |
| 85 | rs17738111 | 5 | 74015025 | 2.09E-04 | 1.99 (1.36-2.90) | 0.168 2.17 (0.82-5.74) | Consistent | 0.024 2.96 (1.21-7.20) | Consistent |
| 86 | rs17556534 | 5 | 155809735 | 2.18E-04 | 2.20 (1.42-3.40) | 0.708 1.76 (0.34-9.21) | Consistent | 0.261 2.71 (0.61-12.07) | Consistent |
| 87 | rs258952 | 5 | 92055195 | 2.20E-04 | 1.62 (1.25-2.09) | 0.498 0.68 (0.27-1.71) | Inconsistent | 0.191 0.56 (0.23-1.32) | Inconsistent |
| 88 | rs1004667 | 14 | 78734765 | 2.20E-04 | 0.61 (0.47-0.79) | 0.180 1.97 (0.83-4.69) | Inconsistent | 0.828 1.17 (0.49-2.77) | Inconsistent |
| 89 | rs6939896 | 6 | 170198941 | 2.20E-04 | 2.09 (1.42-3.06) | 0.804 0.89 (0.33-2.41) | Inconsistent | 0.805 1.16 (0.44-3.08) | Consistent |
| 90 | rs4464906 | 7 | 81273756 | 2.22E-04 | 1.99 (1.39-2.86) | 0.481 0.7 (0.28-1.74) | Inconsistent | 0.819 1.14 (0.46-2.82) | Consistent |
| 91 | rs7109421 | 11 | 21753165 | 2.22E-04 | 0.60 (0.46-0.78) | 0.831 0.89 (0.38-2.11) | Consistent | 0.675 0.83 (0.36-1.92) | Consistent |
| 92 | rs7092350 | 10 | 99140218 | 2.23E-04 | 1.71 (1.28-2.27) | 0.292 1.66 (0.71-3.88) | Consistent | 0.065 2.21 (0.97-5.02) | Consistent |
| 93 | rs7092499 | 10 | 99140313 | 2.23E-04 | 1.71 (1.28-2.27) | 0.292 1.66 (0.71-3.88) | Consistent | 0.065 2.21 (0.97-5.02) | Consistent |
| 94 | rs9888026 | 10 | 99159869 | 2.23E-04 | 1.71 (1.28-2.27) | 0.292 1.66 (0.71-3.88) | Consistent | 0.065 2.21 (0.97-5.02) | Consistent |
| 95 | rs9888117 | 10 | 99159963 | 2.23E-04 | 1.71 (1.28-2.27) | 0.292 1.66 (0.71-3.88) | Consistent | 0.065 2.21 (0.97-5.02) | Consistent |
| 96 | rs12565150 | 1 | 95335020 | 2.24E-04 | 1.82 (1.33-2.51) | 0.827 0.91 (0.38-2.17) | Inconsistent | 0.029 2.69 (1.13-6.38) | Consistent |
| 97 | rs1425725 | 8 | 25600342 | 2.24E-04 | 1.74 (1.30-2.33) | 0.149 1.9 (0.83-4.36) | Consistent | 0.102 2.1 (0.93-4.74) | Consistent |
| 98 | rs10775205 | 15 | 33577145 | 2.25E-04 | 0.61 (0.47-0.80) | 0.012 0.25 (0.09-0.73) | Consistent | 0.001 0.2 (0.08-0.5) | Consistent |
| 99 | rs1990444 | 7 | 69226592 | 2.31E-04 | 2.79 (1.57-4.95) | 0.390 0.59 (0.2-1.79) | Inconsistent | 1.000 1 (0.33-3.07) | Consistent |
| 100 | rs1373469 | 4 | 162220823 | 2.35E-04 | 0.61 (0.47-0.80) | 0.292 0.6 (0.26-1.41) | Consistent | 0.024 0.38 (0.17-0.87) | Consistent |

Abbreviations: OR=odds ratio, CI=confidence interval, SNP=single nucleotide polymorphism, NA=Not available

The list were provided from the original article published by Myung *et al*.

†Physical position based on human reference genome build hg 19 (GRCh37).

‡*P* value and OR were calculated using dominant disease model. The dominant allele is test for the minor allele.

**Supplementary Table S5. Replication of the top 10 SNPs that were reported in our previous genetic study for antidepressant response in Korean population (Lim et al.)**.

| **The top 10 SNPs that were reported in previous genetic study for antidepressant response (Lim et al.)^a^** | | | | | **Quasi-replication for remission in our WGS data^b^** | | | **Replication for response in our WGS data^b^** | | |
| --- | --- | --- | --- | --- | --- | --- | --- | --- | --- | --- |
| **SNP** | **Chro moso me** | **Position** | ***P* value** | **Heterozygote OR (95% CI)** | ***P* value^‡^** | **OR (CI 95%)^‡^** | **Consistency of the direction** | ***P* value^‡^** | **OR (CI 95%)^‡^** | **Consistency of the direction** |
| rs4760815 | 12 | 72372229 | 1.26E-05 | 3.77 (3.55–4.00) | 0.322 | 1.67 (0.64-4.36) | Consistent | 0.328 | 0.59 (0.23-1.54) | Inconsistent |
| rs11179027 | 12 | 72377312 | 1.57E-05 | 2.69 (1.45–4.99) | 0.814 | 1.15 (0.46-2.9) | Consistent | 0.251 | 0.57 (0.23-1.42) | Inconsistent |
| rs543196 | 6 | 102051349 | 4.84E-05 | 1.69 (0.83–3.45) | 0.678 | 1.23 (0.54-2.79) | Consistent | 0.543 | 1.31 (0.58-2.95) | Consistent |
| rs3828275 | 2 | 171682740 | 6.89E-05 | 0.31 (0.17–0.55) | 0.525 | 1.37 (0.58-3.21) | Inconsistent | 0.295 | 1.64 (0.72-3.72) | Inconsistent |
| rs17110532 | 12 | 72364668 | 8.86E-05 | 2.02 (1.14–3.59) | 0.522 | 1.43 (0.62-3.28) | Consistent | 0.837 | 1.11 (0.49-2.53) | Consistent |
| rs2066713 | 17 | 28551665 | 1.26E-04 | 0.48 (0.03–8.42) | 0.651 | 2.33 (0.25-21.71) | Inconsistent | 0.386 | 2.35 (0.37-14.75) | Inconsistent |
| rs572487 | 6 | 102050488 | 1.36E-04 | 1.65 (1.54–1.77) | 0.521 | 1.36 (0.59-3.16) | Consistent | 0.834 | 1.15 (0.50-2.65) | Consistent |
| rs17110747 | 12 | 72425954 | 1.94E-04 | 2.53 (1.37–4.69) | 0.835 | 1.19 (0.53-2.69) | Consistent | 1.000 | 1 (0.45-2.23) | Consistent |
| rs12185692 | 2 | 171670826 | 2.33E-04 | 0.35 (0.20–0.62) | 0.676 | 1.21 (0.52-2.81) | Inconsistent | 0.403 | 1.52 (0.67-3.44) | Inconsistent |
| rs2020942 | 17 | 28546914 | 2.96E-04 | 1.27 (1.21–1.34) | 0.417 | 3.62 (0.42-31.34) | Consistent | 0.433 | 2.11 (0.45-9.99) | Consistent |

Abbreviations: OR=odds ratio, CI=confidence interval, SNP=single nucleotide polymorphism, NA=Not available

The list were provided from the original article published by Lim *et al*.

†Physical position based on human reference genome build hg 19 (GRCh37).

‡*P* value and OR were calculated using dominant disease model. The dominant allele is test for the minor allele.

aThe odds ratio was calculated as the comparison of response group relative to the comparison of non-response group.

bThe odds ratio was calculated as the comparison of non-response (or non-remitted) group relative to the comparison of response (or remitted) group.

**Supplementary Table S6. Replication of the SNPs that were reported in previous meta-analysis of antidepressant response GWAS in European ancestry (Uher et al.).**

| **The SNPs that were reported in previous meta-analysis of antidepressant response GWAS (Uher et al.)^a^** | | | | | **Quasi-replication for remission in our WGS data^b^** | | | **Replication for response in our WGS data^b^** | | |
| --- | --- | --- | --- | --- | --- | --- | --- | --- | --- | --- |
| **SNP** | **Chromosome** | **Position^†^** | **OR** | **p(fixed)** | ***P* value^‡^** | **OR (CI 95%)^‡^** | **Consistency of the direction** | ***P* value^‡^** | **OR (CI 95%)^‡^** | **Consistency of the direction** |
| rs1525293 | 7 | 70473005 | 0.59 | 2.19E-06 | 0.500 | 1.44 (0.6-3.45) | Consistent | 0.514 | 0.73 (0.31-1.73) | Inconsistent |
| rs364477 | 9 | 955794 | 1.69 | 3.96E-06 | 0.404 | 0.65 (0.29-1.48) | Consistent | 0.312 | 1.55 (0.69-3.46) | Inconsistent |
| rs8012941 | 14 | 63322347 | 1.54 | 4.48E-06 | 0.821 | 1.12 (0.46-2.75) | Inconsistent | 0.508 | 1.39 (0.57-3.42) | Inconsistent |
| rs11055387 | 12 | 13519996 | 2.19 | 7.98E-06 | 0.090 | 5.73 (0.7-47.19) | Inconsistent | 1.000 | 1 (0.26-3.79) | Consistent |
| rs2377360 | 1 | 217200883 | 1.60 | 8.77E-06 | 1.000 | 0.98 (0.41-2.33) | Consistent | 0.668 | 0.8 (0.34-1.88) | Consistent |
| rs1034394 | 22 | 34823828 | 1.60 | 1.05E-05 | 0.303 | 0.63 (0.28-1.44) | Consistent | 0.102 | 0.48 (0.21-1.08) | Consistent |
| rs12527253 | 6 | 89293745 | 1.58 | 1.26E-05 | 0.185 | 2.45 (0.75-8.05) | Inconsistent | 0.203 | 0.47 (0.15-1.43) | Consistent |
| rs11761231 | 7 | 131370039 | 1.50 | 1.30E-05 | 0.292 | 0.6 (0.26-1.37) | Consistent | 0.063 | 0.43 (0.18-1) | Consistent |
| rs904759 | 5 | 1736837 | 0.66 | 1.39E-05 | 1.000 | 1.01 (0.43-2.36) | Consistent | 1.000 | 1.08 (0.47-2.48) | Consistent |
| rs234055 | 3 | 172310820 | 0.58 | 1.47E-05 | 0.536 | 0.76 (0.34-1.74) | Inconsistent | 1.000 | 0.97 (0.43-2.17) | Inconsistent |
| rs2233434 | 6 | 44232920 | 0.29 | 1.71E-05 | 0.521 | 0.73 (0.32-1.7) | Inconsistent | 1.000 | 1.04 (0.45-2.37) | Inconsistent |
| rs7032771 | 9 | 120202577 | 0.66 | 1.89E-05 | 0.253 | 4.3 (0.51-36.43) | Consistent | 1.000 | 0.89 (0.2-3.96) | Inconsistent |
| rs2789876 | 9 | 124383244 | 0.68 | 1.94E-05 | 0.700 | 0.73 (0.15-3.48) | Inconsistent | 0.699 | 0.58 (0.11-3.14) | Inconsistent |
| rs1480975 | 4 | 35202323 | 0.63 | 2.02E-05 | 0.306 | 1.85 (0.7-4.92) | Consistent | 0.317 | 1.89 (0.66-5.38) | Consistent |
| rs4683458 | 3 | 142865637 | 2.06 | 2.85E-05 | 0.519 | 0.64 (0.18-2.27) | Consistent | 1.000 | 0.84 (0.23-3.08) | Consistent |
| rs1330656 | 5 | 26048462 | 1.70 | 3.01E-05 | NA | NA | NA | NA | NA | NA |
| rs10091344 | 8 | 34132075 | 1.49 | 3.05E-05 | 0.125 | 2.04 (0.87-4.8) | Inconsistent | 0.289 | 1.64 (0.69-3.9) | Inconsistent |
| rs28602975 | 15 | 40604862 | 0.62 | 3.05E-05 | 1.000 | 0.98 (0.27-3.61) | Inconsistent | 0.340 | 1.94 (0.55-6.85) | Consistent |
| rs12408292 | 1 | 62212822 | 1.47 | 3.31E-05 | 0.827 | 1.16 (0.49-2.73) | Inconsistent | 0.833 | 1.12 (0.48-2.61) | Consistent |
| rs2368266 | 10 | 28144330 | 1.50 | 3.31E-05 | 1.000 | 1.06 (0.47-2.41) | Inconsistent | 0.841 | 1.11 (0.5-2.46) | Consistent |
| rs942659 | 6 | 23784993 | 1.56 | 3.62E-05 | 1.000 | 1.05 (0.46-2.38) | Inconsistent | 0.685 | 0.82 (0.37-1.83) | Inconsistent |
| rs17034196 | 4 | 157116213 | 0.64 | 3.76E-05 | 0.663 | 1.27 (0.53-3.05) | Consistent | 0.389 | 1.56 (0.67-3.6) | Consistent |
| rs2868813 | 7 | 77405741 | 0.69 | 4.22E-05 | 0.536 | 0.76 (0.34-1.74) | Inconsistent | 0.683 | 0.81 (0.36-1.83) | Consistent |
| rs6540437 | 1 | 207823865 | 0.69 | 4.35E-05 | 0.406 | 1.48 (0.64-3.39) | Consistent | 1.000 | 1 (0.45-2.23) | Inconsistent |
| rs4805218 | 19 | 28975361 | 0.70 | 4.72E-05 | 0.600 | 1.38 (0.5-3.82) | Consistent | 0.604 | 0.69 (0.25-1.88) | Consistent |
| rs6984342 | 8 | 81883905 | 0.38 | 4.81E-05 | 0.822 | 1.18 (0.48-2.91) | Consistent | 0.505 | 0.67 (0.27-1.63) | Inconsistent |
| rs7597171 | 2 | 157470262 | 0.64 | 4.95E-05 | 0.021 | 3.14 (1.28-7.69) | Consistent | 0.003 | 4.67 (1.6-13.6) | Consistent |
| rs3803804 | 17 | 1923056 | 1.76 | 4.98E-05 | 0.538 | 0.76 (0.33-1.73) | Consistent | 0.312 | 0.65 (0.29-1.45) | Consistent |
| rs2125000 | 4 | 125265313 | 1.49 | 5.30E-05 | 0.837 | 1.12 (0.49-2.53) | Inconsistent | 1.000 | 1.07 (0.48-2.38) | Inconsistent |
| rs6437137 | 2 | 158980689 | 1.44 | 5.48E-05 | 0.285 | 0.61 (0.26-1.41) | Consistent | 1.000 | 1.04 (0.45-2.37) | Consistent |
| rs88505 | 15 | 70127589 | 1.62 | 6.13E-05 | 0.513 | 0.75 (0.31-1.78) | Consistent | 0.828 | 0.86 (0.36-2.03) | Consistent |
| rs9853121 | 3 | 120272358 | 1.93 | 6.17E-05 | 0.563 | 0.71 (0.23-2.25) | Consistent | 1.000 | 1.15 (0.37-3.6) | Consistent |
| rs2883863 | 14 | 63597544 | 1.48 | 6.44E-05 | 0.834 | 1.11 (0.49-2.56) | Inconsistent | 0.221 | 0.6 (0.26-1.35) | Consistent |
| rs17010751 | 2 | 31374188 | 0.31 | 6.44E-05 | NA | NA | NA | NA | NA | NA |
| rs11752401 | 6 | 18328374 | 0.68 | 6.52E-05 | 0.530 | 0.72 (0.31-1.66) | Inconsistent | 1.000 | 0.93 (0.42-2.09) | Inconsistent |
| rs2516808 | 6 | 168404976 | 1.54 | 7.00E-05 | 0.033 | 0.39 (0.17-0.92) | Consistent | 0.671 | 0.77 (0.33-1.8) | Consistent |
| rs1397831 | 11 | 97632969 | 0.68 | 7.46E-05 | 0.668 | 1.28 (0.54-3.02) | Consistent | 1.000 | 1.04 (0.45-2.37) | Inconsistent |
| rs12744671 | 1 | 68931922 | 1.79 | 7.48E-05 | NA | NA | NA | NA | NA | NA |
| rs10231884 | 7 | 79084800 | 0.70 | 7.53E-05 | 0.375 | 0.64 (0.26-1.59) | Inconsistent | 0.828 | 0.89 (0.38-2.11) | Inconsistent |
| rs17169667 | 7 | 34193952 | 0.37 | 7.56E-05 | 0.294 | 0.27 (0.02-3.08) | Inconsistent | 0.273 | 0.20 (0.01-4.03) | Inconsistent |
| rs12210951 | 6 | 21936868 | 0.64 | 7.79E-05 | 0.551 | 4.15 (0.21-82.73) | Consistent | 0.562 | 3.11 (0.27-35.44) | Consistent |
| rs17510362 | 18 | 9749952 | 0.60 | 7.81E-05 | 0.415 | 2.97 (0.33-26.43) | Consistent | 0.214 | 3.22 (0.56-18.5) | Consistent |
| rs1569448 | 6 | 132437476 | 1.46 | 8.87E-05 | 0.140 | 1.99 (0.87-4.57) | Inconsistent | 0.149 | 1.94 (0.84-4.47) | Inconsistent |
| rs11790991 | 9 | 94873324 | 1.55 | 9.25E-05 | 1.000 | 1.14 (0.27-4.85) | Inconsistent | 1.000 | 1.22 (0.31-4.86) | Inconsistent |
| rs10416595 | 19 | 9102065 | 1.46 | 9.28E-05 | 0.831 | 0.88 (0.38-2.05) | Consistent | 0.208 | 1.77 (0.77-4.04) | Inconsistent |
| rs943347 | 10 | 97278123 | 1.56 | 9.31E-05 | 0.144 | 0.5 (0.22-1.15) | Consistent | 0.014 | 0.35 (0.15-0.79) | Consistent |
| rs11976181 | 7 | 141797564 | 1.57 | 9.37E-05 | 0.793 | 1.27 (0.44-3.71) | Inconsistent | 0.801 | 0.85 (0.3-2.38) | Consistent |
| rs2826852 | 21 | 22836013 | 0.70 | 9.44E-05 | 0.415 | 2.97 (0.33-26.43) | Consistent | 0.036 | 8.43 (0.95-75.11) | Consistent |
| rs1989181 | 6 | 142523865 | 0.61 | 9.62E-05 | 0.532 | 1.37 (0.6-3.11) | Consistent | 0.305 | 1.67 (0.74-3.77) | Consistent |
| rs9866941 | 3 | 183555074 | 0.67 | 9.71E-05 | 0.834 | 1.11 (0.49-2.56) | Consistent | 1.000 | 1 (0.44-2.26) | Consistent |
| rs4363479 | 1 | 202112285 | 2.45 | 9.85E-05 | 1.000 | NA | NA | 1.000 | NA | NA |
| rs4459686 | 2 | 238586642 | 1.50 | 9.85E-05 | 0.297 | 1.67 (0.73-3.84) | Inconsistent | 0.839 | 0.87 (0.39-1.95) | Consistent |
| rs668205 | 9 | 9516348 | 1.43 | 9.94E-05 | 0.822 | 0.84 (0.35-2.03) | Consistent | 0.828 | 1.12 (0.47-2.66) | Inconsistent |
| rs11084396 | 19 | 55865527 | 0.53 | 1.60E-04 | 0.305 | 0.64 (0.28-1.46) | Inconsistent | 0.150 | 0.51 (0.22-1.16) | Inconsistent |

Abbreviations: OR=odds ratio, CI=confidence interval, SNP=single nucleotide polymorphism, NA=Not available

The list were provided from the original article published by Uher *et al*.

*The association results were calculated from our discovery set (n = 100).

†Physical position based on human reference genome build hg 19 (GRCh37).

‡*P* value and OR were calculated using dominant disease model. The dominant allele is test for the minor allele.

aThe odds ratio was calculated as the comparison of remitted (response) group relative to the comparison of non-remitted (non-response) group.

bThe odds ratio was calculated as the comparison of non-remitted (non-response) group relative to the comparison of remitted (response) group.

**Supplementary Table S7 Loss-of-function candidate variants associated with a response following SSRI treatment in patients with MDD (discovery set, *n* = 100; replication set, *n* = 553).**

| **Chromosome** | **SNP** | **Position^†^** | **Gene** | **Minor/**  **Major allele** | **Cohort^a^** | **MAF**  **in non-response** | **MAF**  **in response** | ***p* value^‡^** | **OR (CI, 95%)^‡^** |
| --- | --- | --- | --- | --- | --- | --- | --- | --- | --- |
| 1 | rs115678527 | 152748895 | *LCE1F* | A/C | Discovery | 0 | 0.042 | 0.081 | 0.12 (0.0067-2.32) |
|  |  |  |  |  | Replication | 0.020 | 0.020 | 1 | 0.98 (0.42–2.31) |
|  |  |  |  |  | Combined | 0.017 | 0.024 | 0.435 | 0.71 (0.32–1.57) |
| 9 | rs7021123 | 70918647 | *FOXD4L3* | A/C | Discovery | 0 | 0.050 | 0.078 | 0.10 (0.0057-1.89) |
|  |  |  |  |  | Replication | 0.014 | 0.017 | 0.807 | 0.82 (0.31–2.18) |
|  |  |  |  |  | Combined | 0.012 | 0.022 | 0.201 | 0.52 (0.21–1.29) |
| 9 | rs1476860 | 125391241 | *OR1B1* | A/G | Discovery | 0.450 | 0.317 | 0.096^*^ | 2.61 (0.85–8.04)^*^ |
|  |  |  |  |  | Replication | 0.356 | 0.381 | 0.713^*^ | 1.10 (0.68–1.79)^*^ |
|  |  |  |  |  | Combined | 0.369 | 0.371 | 0.311^*^ | 1.26 (0.81–1.96)^*^ |
| 17 | rs3213755 | 39197499 | *KRTAP1-1* | A/G | Discovery | 0.225 | 0.175 | 0.295 | 1.64 (0.72–3.72) |
|  |  |  |  |  | Replication | 0.362 | 0.241 | 0.00041 | 1.86 (1.32–2.61) |
|  |  |  |  |  | Combined | 0.343 | 0.230 | 0.00018 | 1.83 (1.34–2.51) |

Abbreviations: MAF, minor allele frequency; OR, odds ratio; CI, confidence interval; SNP, single nucleotide polymorphism; NA, not available

^†^Physical position based on human reference genome build hg 19 (GRCh37).

^‡^*p* value and OR were calculated using a dominant disease model.

^*^*p* value was calculated using a recessive disease model.

^a^In the discovery set, response, *n* = 60, non-response, *n* = 40; in the replication set, response, *n* = 299, non-response, *n* = 254.

**Supplementary Table S8 Distribution of samples with minor alleles for considering the dominant disease model in the discovery and the replication set, respectively.**

| SNP (Minor allele) | Discovery set | Replication Set | *P* value*^*^* |
| --- | --- | --- | --- |
| rs115678527 (A) (%) | 5.0 | 4.0 | 0.59 |
| rs7021123 (A) (%) | 6.0 | 3.1 | 0.14 |
| rs1476860 (G) (%) | 59.0 | 60.0 | 0.91 |
| rs3213755 (A) (%) | 38.0 | 44.4 | 0.27 |
| rs139506139 (T) (%) | 3.0 | 3.1 | 1.00 |
| rs877346 (T) (%) | 38.0 | 32.9 | 0.36 |

Abbreviations: SNP, single nucleotide polymorphism

^*^*p* value was calculated using Fisher’s exact test.

**Supplementary Table S9 Gene-based association results applying SKAT-O method in the discover set (*p* < 1.00E-04).**

| **Transcript ID** | **Gene** | **Number of variants** | **SKAT-O *p* value** |
| --- | --- | --- | --- |
| NM_006072 | *CCL26* | 3 | 4.7.E-06 |
| NM_000387 | *SLC25A20* | 6 | 2.6.E-05 |
| NM_014740 | *EIF4A3* | 7 | 4.2.E-05 |
| NM_001301031 | *SLC24A1* | 12 | 4.9.E-05 |
| NM_001301032 | *SLC24A1* | 12 | 4.9.E-05 |
| NM_004727 | *SLC24A1* | 12 | 4.9.E-05 |
| NR_003955 | *EMBP1* | 15 | 5.3.E-05 |
| NM_001254740 | *SLC24A1* | 9 | 5.8.E-05 |
| NM_015157 | *PHLDB1* | 19 | 7.5.E-05 |
| NM_001291732 | *PGAP3* | 10 | 7.6.E-05 |
| NM_001291733 | *PGAP3* | 10 | 7.6.E-05 |
| NM_001291730 | *PGAP3* | 11 | 7.6.E-05 |
| NM_001011515 | *PDLIM5* | 10 | 9.4.E-05 |
| NM_001011516 | *PDLIM5* | 10 | 9.4.E-05 |
